# *GmAMT2.1/2.2*-dependent ammonium nitrogen and metabolites shape rhizosphere microbiome assembly to mitigate cadmium toxicity

**DOI:** 10.1038/s41522-024-00532-6

**Published:** 2024-07-24

**Authors:** Zhandong Cai, Taobing Yu, Weiyi Tan, Qianghua Zhou, Lingrui Liu, Hai Nian, Tengxiang Lian

**Affiliations:** 1 South China Institute for Soybean Innovation Research, College of Agriculture, South China Agricultural University, Guangzhou, Guangdong, China; 2 Guangdong Provincial Key Laboratory of Utilization and Conservation of Food and Medicinal Resources in Northern Region, Shaoguan University, Shaoguan, 512000 China; 3 Guangdong Provincial Key Laboratory for the Development Biology and Environmental Adaptation of Agricultural Organisms, South China Agricultural University, Guangzhou, Guangdong China; 4 Key Laboratory for Enhancing Resource Use Efficiency of Crops in South China, Ministry of Agriculture and Rural Affairs, South China Agricultural University, Guangzhou, Guangdong China

**Keywords:** Applied microbiology, Microbial communities

## Abstract

Cadmium (Cd), a heavy metal, is negatively associated with plant growth. AMT (ammonium transporter) genes can confer Cd resistance and enhance nitrogen (N) uptake in soybeans. The potential of AMT genes to alleviate Cd toxicity by modulating rhizosphere microbiota remains unkonwn. Here, the rhizosphere microbial taxonomic and metabolic differences in three genotypes, i.e., double knockout and overexpression lines and wild type, were identified. The results showed that *GmAMT2.1/2.2* genes could induce soybean to recruit beneficial microorganisms, such as *Tumebacillus*, *Alicyclobacillus*, and *Penicillium*, by altering metabolites. The bacterial, fungal, and cross-kingdom synthetic microbial communities (SynComs) formed by these microorganisms can help soybean resist Cd toxicity. The mechanisms by which SynComs help soybeans resist Cd stress include reducing Cd content, increasing ammonium (NH_4_^+^-N) uptake and regulating specific functional genes in soybeans. Overall, this study provides valuable insights for the developing microbial formulations that enhance Cd resistance in sustainable agriculture.

## Introduction

Cadmium (Cd) is a ubiquitous heavy metal with a widely distributed and known toxicity^1^, and contamination of agricultural land by Cd is primarily caused by human activities, such as industrial wastewater discharge, disposal of large quantities of metal wastes and sewage sludge, and pesticide misuse^2^. Cd is more mobile than other heavy metals in plants, leading to its increased accumulation and subsequent induction of severe toxicity^3,4^. This is caused by cation deficiency, inhibition of the biosynthesis of chlorophyll or other essential substances, and increased oxidative damage, such as structural and functional cellular degeneration and destruction of biomolecules, which are closely linked to plant growth and development^5^.

The composition and functional activities of the plant rhizosphere microbiome are dynamically regulated under various environmental stresses, providing a critical foundation for plant adaptation and health^6–9^. Interestingly, some microorganisms can reduce the toxicity of Cd to plants and themselves by adsorbing, complexing, enzymatically converting, and redox-converting Cd ions^10,11^. For instance, *Klebsiella mobilis CIAM 880* has the ability to form complexes with unbound Cd ions, which reduces their bioavailability of Cd to barley plants and mitigates the toxic effects^12^. *Enterobacter bugandensis* can also reduce Cd accumulation in wheat grains by bioprecipitation and extracellular adsorption^9,13^.

Plant functional genes, including those involved in biotic and abiotic stress response and nutrient uptake and transport, could modulate the secretion of root exudates and some ionic signals, which significantly influence the rhizosphere microorganisms and are essential for enhancing plant stress tolerance and nutrient use efficiency^14–17^. Conversely, the activity of rhizosphere microorganisms can induce systemic tolerance in plants by releasing metabolites, which can affect host genes expression, and alter phytohormone secretion^17,18^. For example, Pseudomonas aeruginosa and Burkholderia gladioli are able to reduce Cd uptake and expression of metal transporter genes in tomatoes plants, leading to improved growth and photosynthetic pigmentation^19^. *NRT1.1B*, encoding a nitrate transporter in rice, is involved in the attraction of bacteria that are enriched in *O. sativa subsp. indica rice*. These bacteria can improve rice growth under organic nitrogen (N) conditions using the synthetic community^20^. Our recent work has shown that loss of *sst* gene function in rice can affect rhizosphere microbes by altering plant metabolites, such as salicin and arbutin, which in turn can help the host to resist salt stress^21^.

Ammonium transporters (AMTs), involved in ammonium (NH_4_^+^-N) uptake and transport in most plants, promote the uptake of N sources by plants^22,23^. Previous studies have investigated the role of AMTs in promoting NH_4_^+^-N transport to alleviate salt stress^24^. A growing body of evidence suggests that AMTs-targeted NH_4_^+^-N is more sensitive to Cd concentration than NO_3_^-^-N. For example, NH_4_^+^-N can inhibit Cd translocation from roots to shoots, thereby protecting *Arabidopsis thaliana* from Cdtoxicity^25,26^. Recent research^27^ has also found in *Solanum nigrum L*. that the transcription level of Cd transport-related genes is regulated by NH_4_^+^-N signaling to prevent the Cd accumulation and flux, compared to NO_3_^-^-N. The researchers also suggest that NH_4_^+^-N can fix Cd in the cell wall component, thereby improving Cd tolerance. However, the potential of *GmAMT2.1/2.2* genes to alleviate external Cd toxicity by regulating metabolites and microbiota in the soybean rhizosphere remains to be determined.

Synthetic communities (SynComs) have been increasingly used to study the interactions between microbes and their hosts in recent years^28,29^. The development of high-throughput microbial isolation techniques has enabled researchers to efficiently isolate more microbial strains^30^. The isolated strains were then compared with the sequencing results to select suitable candidates for SynComs construction. Despite the fact that research on SynComs has often focused on bacteria, fungi also play a very important role in helping plants resist heavy metal stress^29^. In addition, fungi can establish symbiotic networks with bacteria^31^, which may be more effective in helping plants resist stresses. Therefore, it is necessary to evaluate the role of different synthetic communities in helping soybeans resist Cd toxicity.

In this study, we hypothesized that the *GmAMT2.1/2.2* genes enhance NH_4_^+^-N uptake in soybeans, and help them to resist Cd toxicity by influencing metabolites to recruit beneficial rhizosphere microorganisms. To test these hypotheses, high-throughput sequencing, and liquid chromatography-mass spectrometry (LC-MS) assays were used to identify taxonomic and metabolic differences and their correlations in the rhizosphere. In addition, we isolated and identified bacteria and fungi in the rhizosphere of different genotypes and constructed bacterial, fungal, and bacterial-fungal cross-kingdom SynComs. Finally, we verified that these SynComs influence the expression of heavy metal tolerance-related genes in soybean roots. The objectives of this study were to: (1) investigate how the *GmAMT2.1/2.2* genes influence rhizosphere microbes by regulating metabolites and N patterns, which in turn help soybean to resist Cd toxicity; and (2) determine which SynCom consisting of recruited microorganisms is most effective in alleviating Cd toxicity in soybean, and to elucidate the corresponding physiological and molecular mechanisms.

## Result

### *GmAMT2.1/2.2* are responsible for the Cd toxicity alleviation through affecting N patterns in soybean

**Fig. 1:**
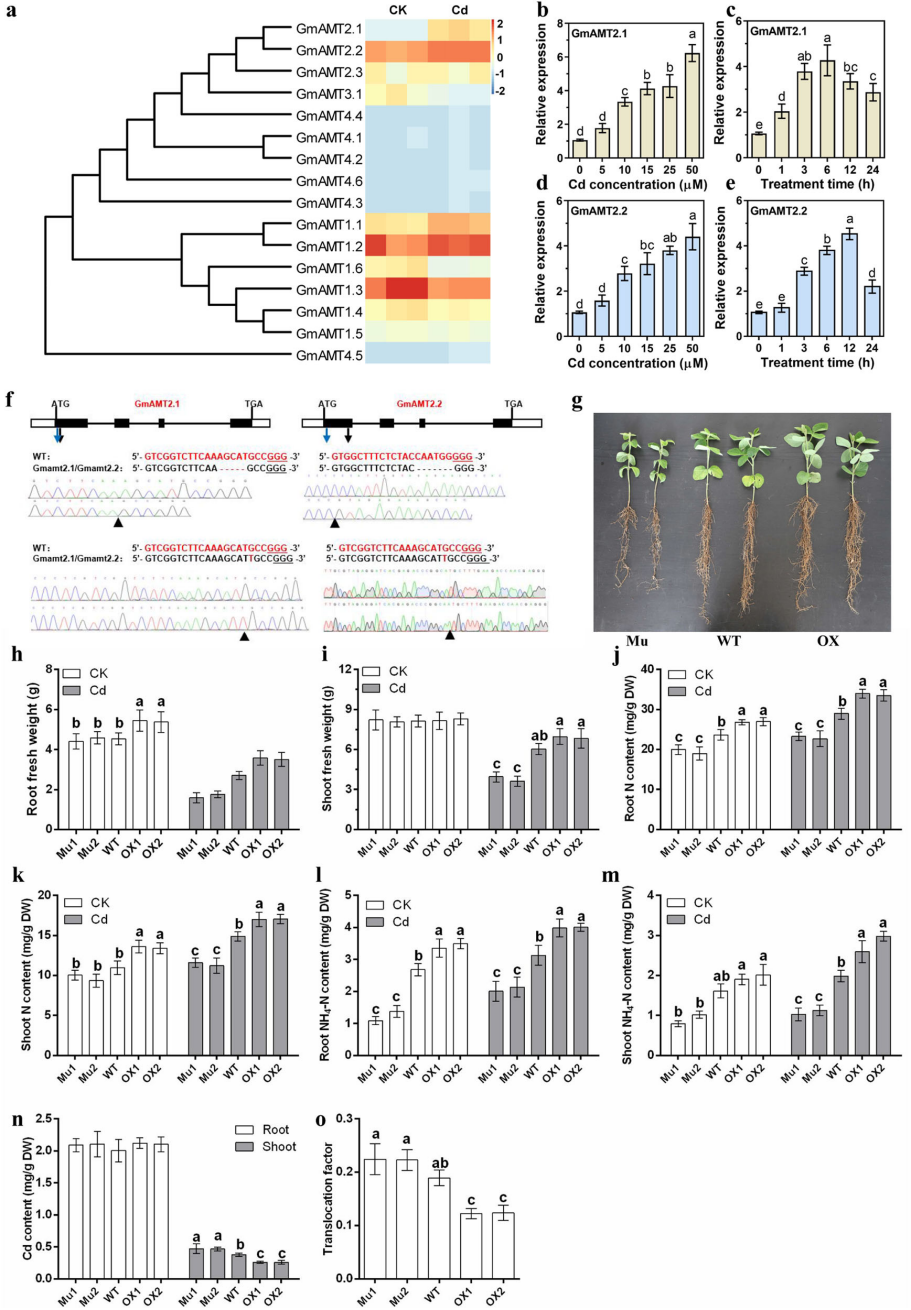
*GmAMT2.1* and *GmAMT2.1* are responsible for alleviating Cd toxicity in soybean by modulating N patterns. **a** Heatmap of the expression of the AMT gene family in soybean under Cd toxicity. Expression pattern analysis of *GmAMT2.1* (**b** and **c**) and *GmAMT2.2* (**d** and **e**). **f** CRISPR/Cas9 mediated gene editing of *GmAMT2.1* and *GmAMT2.2* in soybean. **g** The growth performance of Mu, WT and OX lines under Cd toxicity. The root fresh weight (**h**), shoot fresh weight (**i**), root N content (**j**), shoot N content (**k**), root NH_4_^+^-N content (**l**), shoot NH_4_^+^-N content (**m**), and Cd content (**n**) of Mu, WT and OX lines under CK and Cd conditions. **o** translocation factor of Mu, WT and OX lines under Cd toxicity. Different letters represent significant differences (one-way ANOVA, *n* = 6, *P* < 0.05). The error bars on the columns represent the standard errors (SE). Mu: *GmAMT2.1/2.2* double knockout lines; WT: wild type; OX: *GmAMT2.2* overexpression lines; Cd: cadmium toxicity.

To identify potential candidate AMTs in response to the Cd toxicity alleviation, we examined the transcriptome data of AMT homologous genes in the CK and Cd treatment. Among these, two homologous genes *GmAMT2.1/2.2*, which are predominantly expressed in roots (Supplementary Fig. 1), are significantly up regulated by Cd treatment (Fig. 1a). To further explore the transcriptional response of *GmAMT2.1/2*.2 to Cd treatment, we analyzed the expression levels of *GmAMT2.1/2*.2 using the soybean plants treated with Cd at different concentrations. The expression levels of *GmAMT2.1/2*.2 exhibited an upward trend with increasing Cd concentration and time (except for a decrease in *GmAMT2.1* expression after 6 h of treatment and a decrease in *GmAMT2.2* expression after 12 h of treatment) (Fig. 1b–e). In addition, we generated four lines to confirm the identification and function of *GmAMT2.1/2.2*, including two stable double knockout lines (GmAMT2.1/2.2) and two overexpression lines (OXAMT2.2). Mu1 deleted 5 bp (AGCAT) in sgRNA1 of *GmAMT2.1*, and deleted 7 bp (CAATGGG) in sgRNA2 of *GmAMT2.2*. Mu2 inserted 1 bp (T) in sgRNA1 of both *GmAMT2.1* and *GmAMT2.2*, which ultimately lead to Mu1 and Mu2 are loss-of-function mutants. In the two overexpressed lines (OX1 and OX2), the expression of *GmAMT2.2* was up-regulated 1.87- and 2.07-fold, respectively. (Fig. 1f; Supplementary Figs. 1, 2).

When exposed to identical Cd concentrations, the knockout lines consistently displayed a more pronounced Cd sensitivity compared to the wild-type (WT) lines in terms of plant growth. In contrast, the overexpressed lines did not show significant differences compared to the WT (Fig. 1g). Cd treatment resulted in a significant decrease in the fresh weight of all four lines. Furthermore, an increase in N content, especially NH_4_^+^-N, was observed in response to Cd toxicity. This upward trend in N content progression was also observed upon overexpression of *GmAMT2.2* as well, whereas an opposite relationship was observed in the mutants (Fig. 1h–o). Taken together, these findings suggest that NH_4_^+^-N may play a role in alleviating Cd toxicity and that the *GmAMT2.1/2.2* genes may inhibit Cd transport and accumulation by increasing NH_4_^+^-N levels, thereby mitigating the detrimental effects of Cd toxicity on soybean plants.

### Modification of rhizosphere microbiota by *GmAMT2.1/2.2* under Cd toxicity

**Fig. 2:**
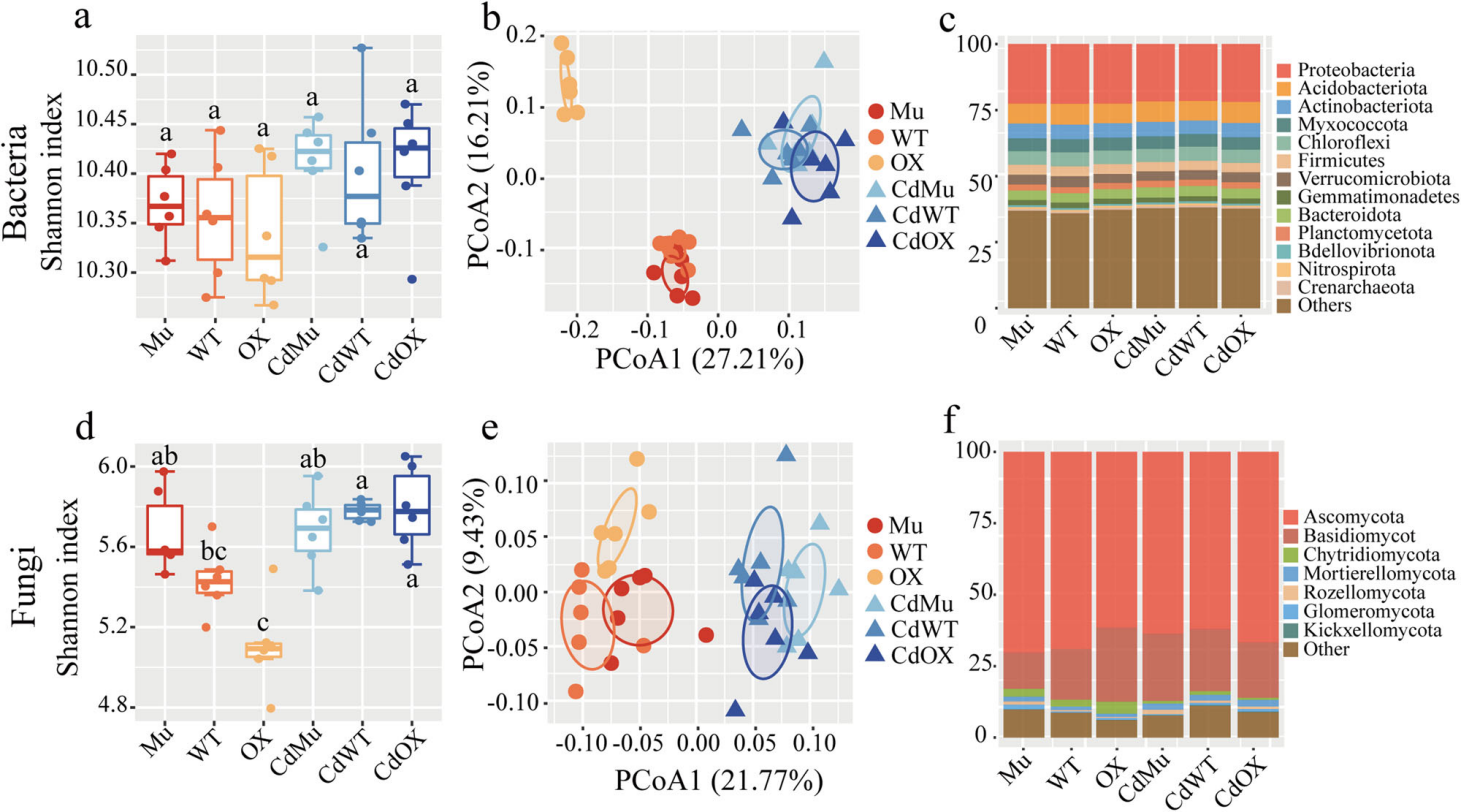
Effect of Cd toxicity on different genotypes of soybean rhizosphere microorganisms. Shannon diversity index of (**a**) bacterial and (**d**) fungal communities (one-way ANOVA, *n* = 6, *P* < 0.05). The Bray-Curtis’s similarity-based Principal Coordinate Analysis (PCoA) showed dissimilarities in the composition of (**b**) bacterial and (**e**) fungal communities (PERMANOVA, *n* = 6, *P* < 0.05). The relative abundances of the (**c**) bacterial and (**f**) fungal phyla. Mu: *GmAMT2.1/2.2* double knockout lines; WT: wild type; OX: *GmAMT2.2* overexpression lines; Cd: cadmium toxicity.

From 2,236,825 high-quality bacterial 16 S rRNA reads and 2,078,640 fungal ITS reads, we identified a total of 11,980 bacterial OTUs and 1,702 fungal OTUs. Cd toxicity had no significant effect (*P* > 0.05) on the alpha diversity of rhizobacterial communities, but increased the alpha diversity of fungal communities in both WT and OX soybean genotypes. Under no-Cd toxicity conditions, the Mu genotype had a higher Shannon index than the other two genotypes, while Cd toxicity did not result in a significant difference between the three genotypes (Fig. 2a, d). In terms of beta diversity, Cd toxicity and the interaction of Cd toxicity and genotype significantly altered the structure of microbial communities (Fig. 2b, e; Supplementary Table 1). Furthermore, WT, OX, and Mu genotypes generally harbored significantly different microbial communities under both Cd and no-Cd toxicity conditions (PERMANOVA, pairwise comparison, *n* = 6, *P* < 0.05) (Fig. 2c, d; Supplementary Table 1), suggesting that the *GmAMT2.1/2.2* gene activity affects the assembly of the soybean rhizosphere microbiome.

The rhizosphere soil of the three genotypes exhibited comparable community composition under Cd and no-Cd toxicity conditions (Fig. 2e, f). Briefly, the most prevalent bacterial phyla were Acidobacteria, Proteobacteria, Actinobacteria and Chloroflexi with the relative abundances ranging from 21.56% to 22.65%, 7.42% to 7.98%, 5.09% to 5.55% and 4.98% to 5.40%, respectively. The fungal communities were mainly Ascomycota, Basidiomycota and Chytridiomycota, with the relative abundances varying from 62.08% to 70.27%, 12.71% to 25.92% and 0.73% to 4.26%, respectively. In general, five bacterial phyla (i.e., Proteobacteria, Actinobacteria, Myxococcota, Firmicutes and Gemmatimonadetes) and five fungal phyla (i.e., Ascomycota, Basidiomycota, Chytridiomycota, Mortierellomycota and Kickxellomycota) were significantly affected by Cd toxicity. One bacterial phylum (i.e., Verrucomicrobiota) and one fungal phylum (i.e., Basidiomycot) were influenced by genotypes. Moreover, three bacterial (i.e., Acidobacteriota, Actinobacteriota and Verrucomicrobiota) and three fungal phyla (i.e., Ascomycota, Basidiomycot and Glomeromycota) were altered by the interactions between genotype and Cd toxicity (Supplementary Table 3).

**Fig. 3:**
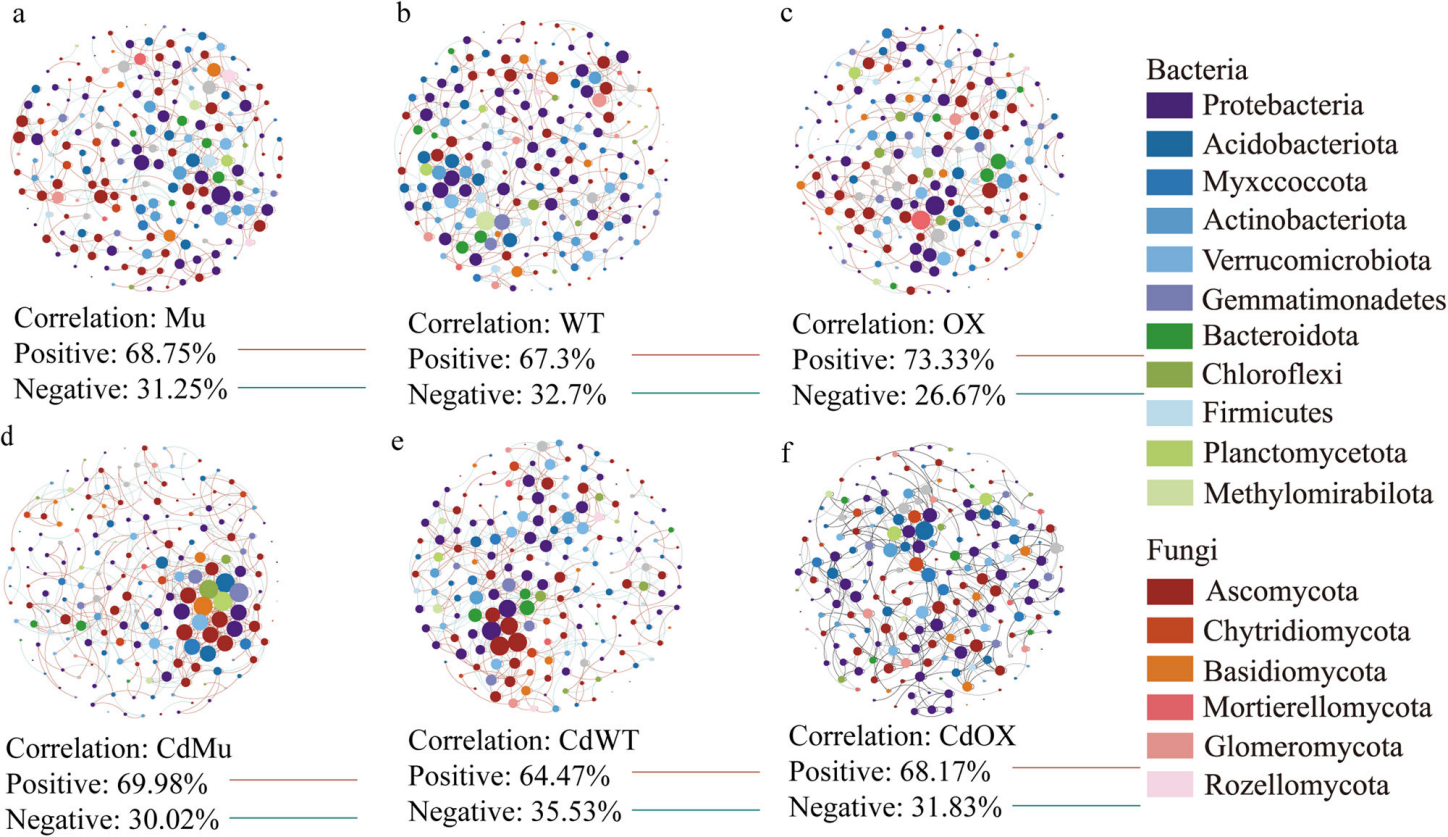
Effect of Cd toxicity on the network structure of rhizosphere microorganisms in different genotypes of soybean. The structure of co-occurrence network of rhizosphere microbial community for (**a**) Mu, (**b**) WT, (**c**) OX, (**d**) CdMu, (**e**) CdWT and (**f**) CdOX. The nodes are colored based on the bacterial and fungal phyla. The edges between nodes indicate significant positive (red) or negative (green) correlation (Spearman’s r > 0.8 or r < -0.8, *P* < 0.01). Mu: *GmAMT2.1/2.2* double knockout lines; WT: wild type; OX: *GmAMT2.2* overexpression lines; Cd: cadmium toxicity.

All genotypes were observed to be significantly affected by Cd toxicity in terms of the co-occurrence network structure, but the responses varied (Fig. 3; Supplementary Table 3). Specifically, Cd toxicity increased network complexity, as evidenced by an increase in both the clustering coefficient and the number of edges. Mu genotype showed the highest number of edges and clustering coefficient under Cd toxicity, followed by WT and OX genotypes. These results underline the impact of Cd toxicity on microbial community dynamics and the genotype-specific mechanisms that shape community composition under Cd toxicity.

### Enriched potential beneficial microorganisms by *GmAMT2.1/2.2* under Cd toxicity

**Fig. 4:**
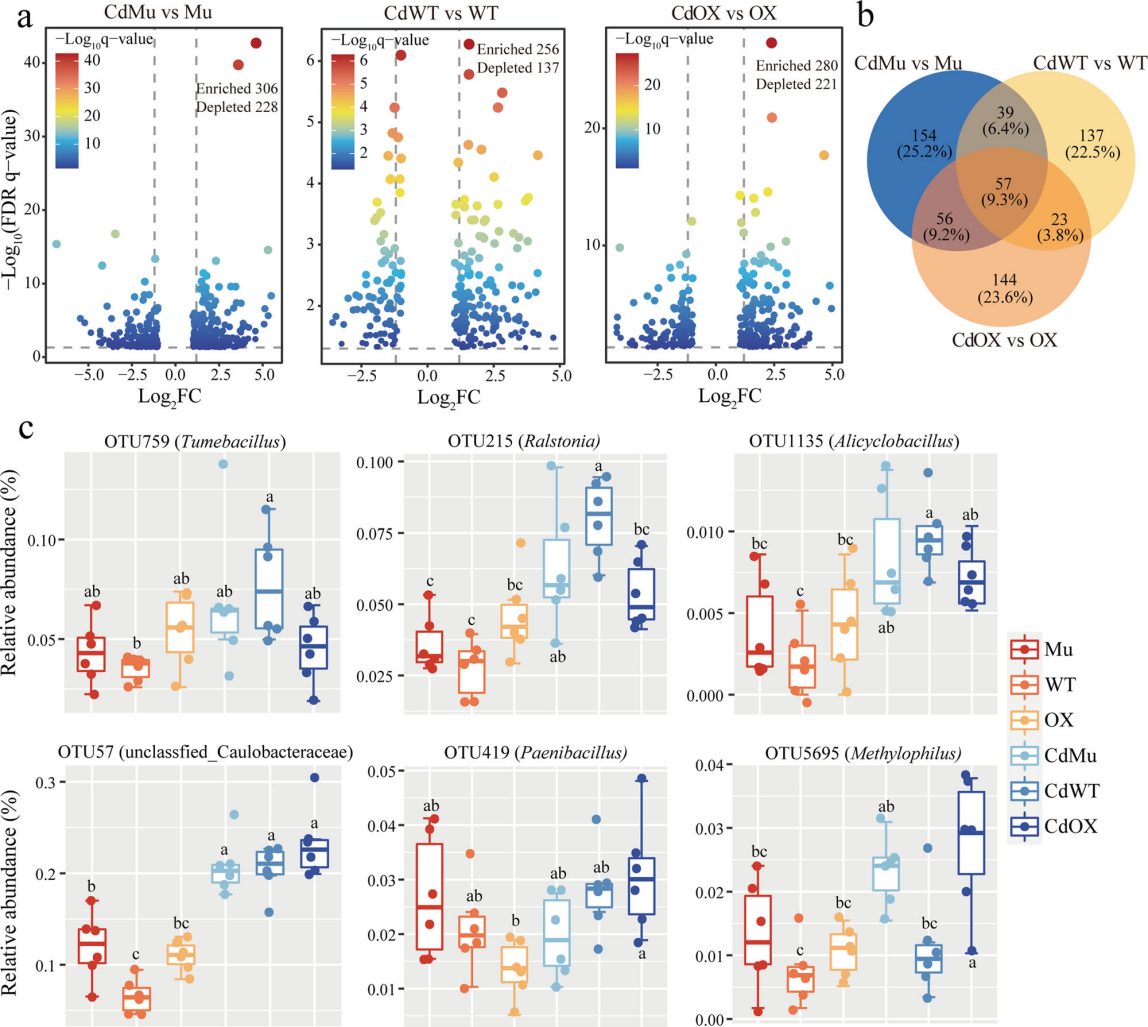
Effects of Cd toxicity on rhizosphere bacterial OTUs of different soybean genotypes. Volcano plots depicting enrichment and depletion bacterial OTUs in (**a**) Mu, WT and OX plants under Cd toxicity compared to no Cd toxicity conditions. **b** Venn analysis showing the unique and shared enriched OTUs between genotypes and Cd toxicity. **c** Relative abundance of six bacterial OTUs in different treatments that significant enriched in the rhizosphere. Mu: *GmAMT2.1/2.2* double knockout lines; WT: wild type; OX: *GmAMT2.2* overexpression lines; Cd: cadmium toxicity.

**Fig. 5:**
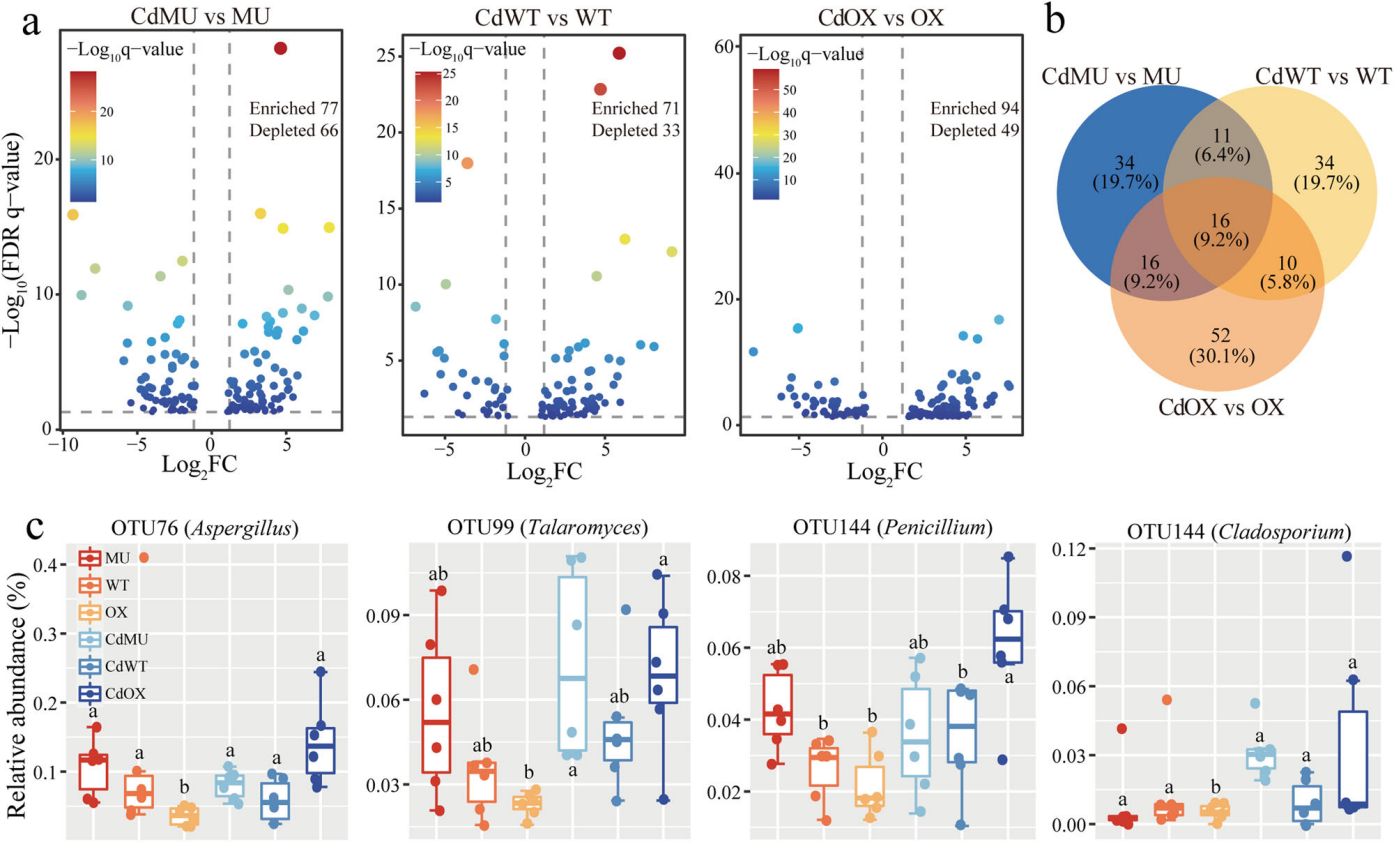
Effects of Cd toxicity on rhizosphere fungal OTUs of different soybean genotypes. Volcano plots depicting enrichment and depletion bacterial OTUs in (**a**) Mu, WT and OX plants under Cd toxicity compared to no Cd toxicity conditions. **b** Venn analysis showing the unique and shared enriched OTUs between genotypes and Cd toxicity. **c** Relative abundance of four fungal OTUs in different treatments that significant enriched in the rhizosphere. Mu: *GmAMT2.1/2.2* double knockout lines; WT: wild type; OX: *GmAMT2.2* overexpression lines; Cd: cadmium toxicity.

We investigated the effect of Cd toxicity on the relative abundance of microbes in three soybean genotypes (Figs. 4, 5). Cd toxicity increased the number of microbial OTUs in Mu, WT and OX by 306, 256, and 280 (bacteria), and 77, 71, and 94 (fungi), respectively (Figs. 4b, 5b). The enriched bacterial OTUs were mainly belonged to Proteobacteria, Firmicutes, Chloroflexi, and Verrucomicrobiota, while the enriched fungal OTUs were mainly associated with Ascomycota and Rozellomycota. Venn analysis revealed that WT and OX genotypes carrying the AMT genes showed a specific enrichment of 274 bacterial and 96 fungal OTUs under Cd toxicity. Based on the information of the isolation and identification of culturable microbes, six bacterial OTUs including OTU759 (*Tumebacillus*), OTU215 (*Ralstonia*), OTU1135 (*Alicyclobacillus*), OTU57 (*Burkholderia*), OTU419 (*Paenibacillus*), OTU5695 (*Methylophilus*), and six fungal OTUs including OTU76 (*Aspergillus*), OTU99 (*Talaromyces*), OTU144 (*Penicillium*) and OTU168 (*Cladosporium*) were identified and selected to show their relative abundance in different treatments (Figs. 4c, 5c).

### Response of soil metabolites to *GmAMT2.1/2.2* under Cd toxicity

**Fig. 6:**
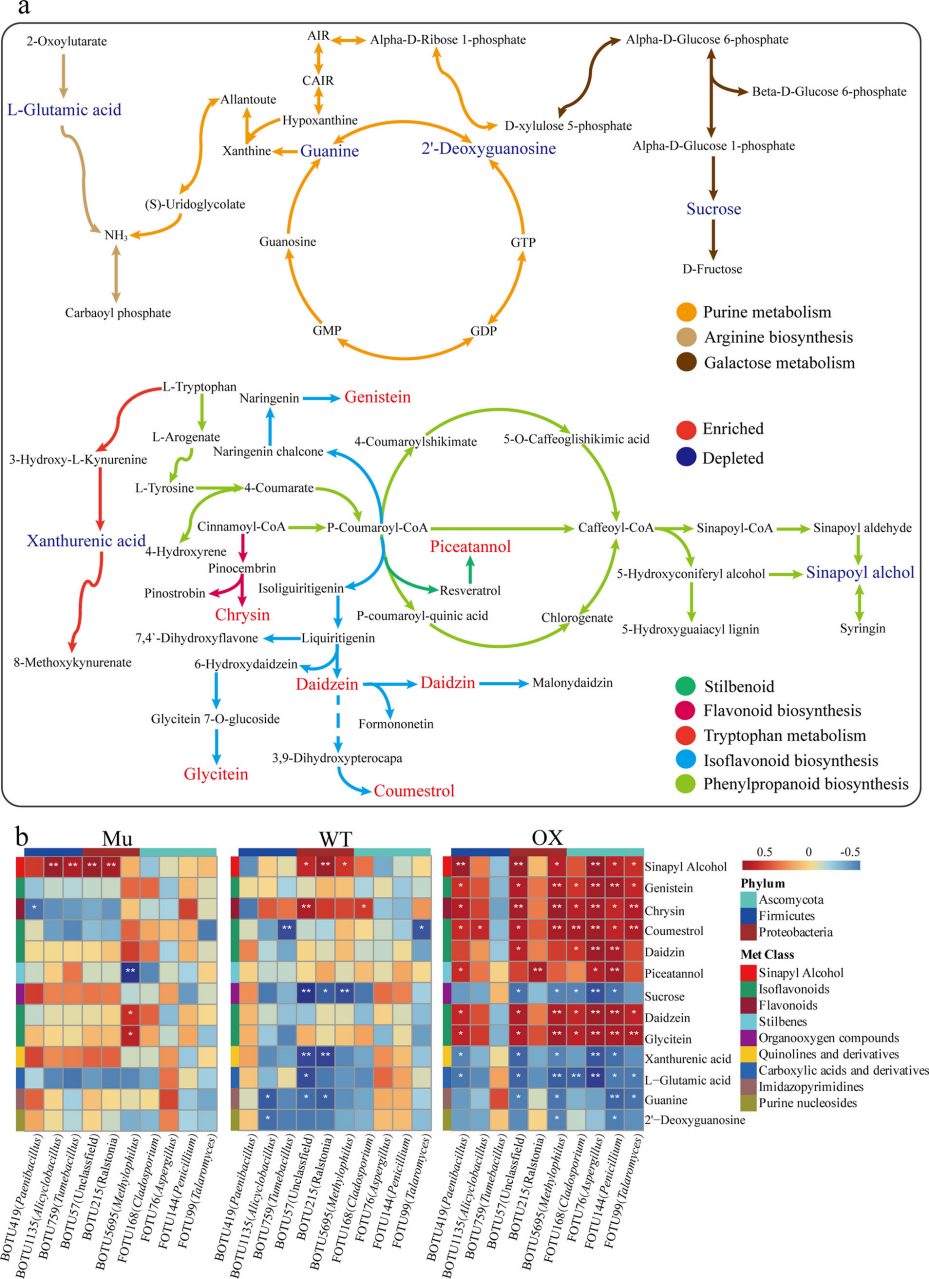
Effect of Cd toxicity on metabolites and metabolite-microbe correlations. **a** The process involves screening for metabolic pathway maps related to crucial differentially expressed metabolites. The red color indicates the metabolites that were increased in plants with *GmAMT2.1/2.2* gene under Cd toxicity, while the green color indicates the metabolites that were decreased. **b** Effect of Cd toxicity on metabolite-microbe correlations in the rhizosphere soil of Mu, WT and OX. Mu: *GmAMT2.1/2.2* double knockout lines; WT: wild type; OX: *GmAMT2.2* overexpression lines; Cd: cadmium toxicity.

To explore the complex metabolic alterations in the rhizosphere soil under different treatments and genotypes, we performed LC-MS analysis on soil metabolites of Mu, WT, and OX with and without Cd exposure. A total of 798 distinct peaks with defined identities were detected across the three genotypes. Orthogonal partial least squares discriminant analysis (OPLS-DA) illustrated a clear separation between the treatments (Supplementary Fig. 4; Supplementary Table 4). Using a combination of filtering procedures and a threshold of variable importance in projection (VIP) > 1.0 and *P* < 0.05, we identified 35 Cd-induced metabolites that increased and 30 that decreased in OX and/or WT, respectively (Supplementary Table 5). KEGG pathway enrichment analysis revealed differential enrichment and depletion of metabolites in secondary metabolism biosynthesis, including purine metabolism, arginine biosynthesis, galactose metabolism, stilbenoid, flavonoid biosynthesis, tryptophan metabolism, isoflavonoid biosynthesis, and phenylpropanoid biosynthesis (Fig. 6a). Notably, the levels of genistein, chrysin, piceatannol, glycitein, daidzein, daidzin, and coumestrol increased, while xanthurenic acid, sinapoyl alcohol, L-glutamic acid, guanine, 2-deoxyguanosine and sucrose decreased in WT and/or OX plants under Cd exposure (Fig. 6a). Correlation analysis between metabolites and microbial species revealed a positive association between sinapyl Alcohol, daidzein, and glycitein with *Alicyclobacillus*, *Tumebacillus*, *Ralstonia*, and *Methylophilus* in Mu and WT genotypes. However, a significant increase in the levels of these metabolites in the OX genotypewas positively correlated with the abundance of different microbial species (Fig. 6b). These findings suggest that *GmAMT2.1/2.2* may play a role in regulating metabolic pathways associated with microbial community assembly in the rhizosphere.

### Effect of different SynComs on Cd tolerance in soybean

**Fig. 7:**
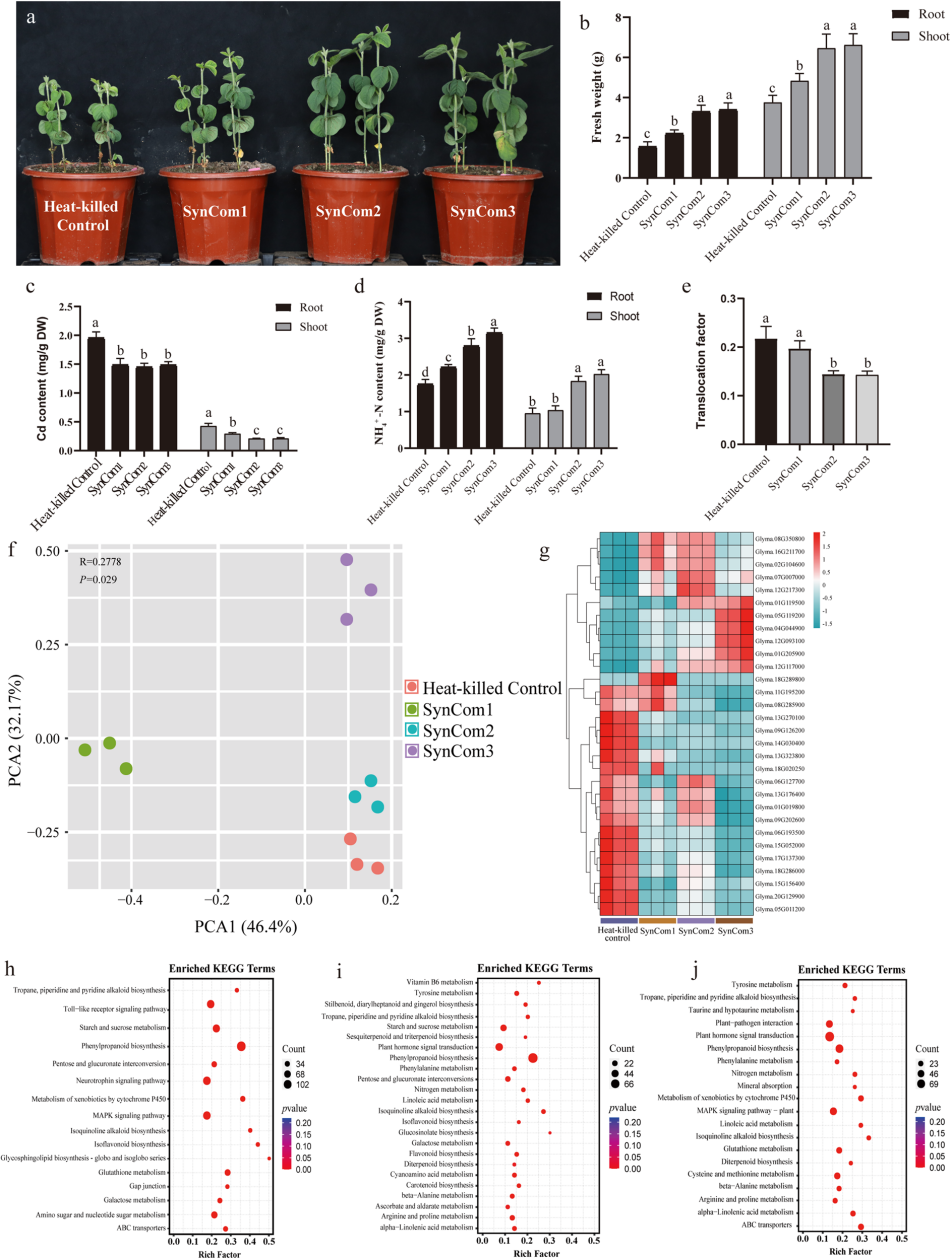
Effect of three SynComs on soybean gene transcription and their regulatory effects. Effect of different SynComs (SynComs1: bacterial SynComs; SynComs2: fungal SynComs; SynComs3: cross-kingdom SynComs) on (**a**) soybean plant growth (**b**) fresh weight, (**c**) Cd content in soybean root, (**d**) NH_4_^+^-N content and (**e**) translocation factor under Cd toxicity. Soybean plants were grown in sterile soil and inoculated with SynComs or heat-killed SynComs. Soybean plants were grown in sterile soil, inoculated with SynComs or heat-killed SynComs. **f** Principal component analysis (PCA) showed distinctions in gene expression of Mu genotype plants between the three SynComs and heat-killed control treatments. **g** Heatmap with hierarchical clustering analysis showing the differential DEGs between the three SynComs and heat-killed control treatments. KEGG analysis of DEGs in response to (**h**) bacterial, (**i**) fungal, and (**j**) cross-kingdom SynComs inoculation under Cd toxicity. Dot size and color indicate the number of DEGs and p-value, respectively. The error bars on the columns represent the standard errors (SE).

Using a high-throughput cultivation method, we sequenced and identified 403 bacterial and 268 fungal colonies from the rhizosphere compartment across different treatments. Among them, six bacterial and eight fungal strains, identical to the microorganisms enriched in the soybean genotypes carrying the AMT genes based on the sequencing results, were used to construct three SynComs (bacterial, fungal, and cross-kingdom SynComs) (Supplementary Table 6). The efficacy of these SynComs in enhancing soybean Cd toxicity tolerance was evaluated (Fig. 7). The results showed that SynComs-treated plants had significantly higher shoot and root fresh weight, NH_4_^+^-N content, and translocation factor, with reduced Cd content in shoot and root compared to heat-killed control plants (Fig. 7a–d). Additionally, cross-kingdom and fungal SynComs had more pronounced impact on the indicators mentioned above compared to bacterial SynCom.

RNA-Seq analysis was conducted to elucidate the molecular mechanisms underlying the SynCom-mediated enhancement of soybean growth under Cd toxicity. PCA showed distinct separation of the four treatments (*P* < 0.05) (Fig. 7f). SynComs application induced differential expression of numerous genes in soybean root tissues (Fig. 7g). Notably, all three SynComs significantly upregulated the expression of genes involved in cytochrome P450 (*Glyma.08G350800*), endopeptidase inhibitor activity (*Glyma.16G211700*), metabolic process related (*Glyma.07G00700*0, *Glyma.01G205900* and *Glyma.02G104600*), zinc finger protein (*Glyma.04G044900*), messenger RNA biogenesis (*Glyma.12G093100*), ethylene response factor (Glyma.12G117000) and BURP domain family member (*Glyma.12G217300*). Bacterial SynCom specifically upregulated genes related to cell redox homeostasis and casparian strip membrane domain proteins (CASP) family member, while fungal SynCom exclusively upregulated genes involved in protein phosphorylation. KEGG enrichment analysis was further performed to explore the distinct metabolic pathways modulated by the three SynComs in alleviating Cd toxicity (Fig. 7h–j). The results showed that the three SynComs mainly influenced metabolic processes associated with amino acid metabolism, biosynthesis of other secondary metabolites, carbohydrate metabolism and signal transduction. Among these, phenylpropanoid biosynthesis exhibited the highest enrichment in both bacterial and fungal SynComs, while plant hormone signal transduction showed the highest enrichment in cross-kingdom SynCom. However, certain pathways demonstrated specific enrichment under different SynCom treatments. For instance, the toll-like receptor signaling pathway was specially enriched in bacterial SynCom treatment and linked to plant disease resistance and immune response. Flavonoid biosynthesis, ascorbate and aldarate metabolism and glucosinolate biosynthesis were exclusively associated with fungal SynCom, while plant-pathogen interaction and mineral absorption were only associated with cross-kingdom SynCom.

## Discussion

**Fig. 8:**
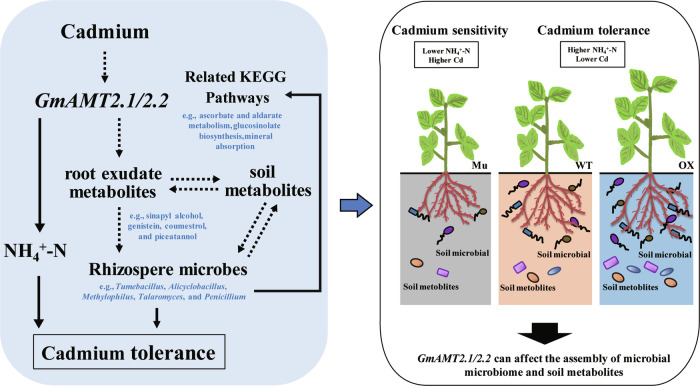
A schematic model summarizing the hypothesized mechanisms of how GmAMT2.1/2.2 confers soybean Cd resistance and plant growth. Soybean under Cd toxicity induces the GmAMT2.1/2.2, leading to the recruitment of beneficial microorganisms, such as *Tumebacillus*, *Alicyclobacillus*, *Methylophilus*, *Aspergillus*, *Talaromyces*, and *Penicillium*, by altering metabolites such as sinapyl alcohol, genistein, coumestrol, and piceatannol. SynComs composed of these specific microorganisms enhance soybean resistance to Cd toxicity by collectively influencing various molecular mechanisms, including ascorbate and aldarate metabolism, glucosinolate biosynthesis, plant-pathogen interactions, and mineral absorption pathways, with cross-kingdom and fungal SynComs demonstrating significant synergistic effects.

Plant functional genes can actively shape the microbial community structure of the rhizosphere by regulating root metabolites, which improving the nutrient uptake efficiency and environmental stress resistance of crops^14,15,29^. AMTs regulate the uptake and transport of NH_4_^+^-N, thereby promoting plant uptake of N sources^24^. NH_4_^+^-N produces various N-containing macromolecules, which regulate Cd uptake and translocation by plants, and alter the soybean root exudate and rhizosphere environment^32,33^. In this study, we used three soybean genotypes with normal expression (WT), overexpression (OX) and knockout (Mu) of the *GmAMT2.1/2.2* genes, respectively, to demonstrate the key role of the *GmAMT2.1/2.2* genes in influencing NH_4_^+^-N uptake, Cd levels, and soil metabolites, resulting in significant shifts in rhizosphere microbial community structure (Fig. 8).

Plant materials carrying the *GmAMT2.1/2.2* genes have been found to attract a diverse array of potentially beneficial microorganisms, including bacterial species such as *Tumebacillus*, *Alicyclobacillus*, *Paenibacillus*, and *Methylphilus*, and fungal species such as *Aspergillus*, *Penicillium*, *Cladosporium*, and *Talaromyces* (Figs. 4, 5). The enriched microbial species with relevant function may play an essential role in enhancing plant resilience to Cd toxicity. For instance, *Tumebacillus* and *Alicyclobacillus* are soil organic matter (SOM) decomposers that can mobilize scarce nutrients, thereby improving plant adaptation to Cd toxicity^34–36^. *Paenibacillus* and *Methylphilus* have been demonstrated to promote plant N uptake^37,38^. *Talaromyces* sp., a plant growth-promoting fungus, releases terpenoid-like volatiles that stimulate plant growth^39^. Certain *Ralstonia* species, such as *Ralstonia eutropha Q2-8* and *Ralstonia Bcul-1*, are considered heavy metal-resistant strains and can enhance plant Cd tolerance through different mechanisms, including modulating heavy metal resistance gene expression, strengthening cell wall components, or sequestering Cd into root cell vacuoles^40,41^. *Aspergillus* sp. activated genes encoding glutathione (GSH) under Cd toxicity, alleviating the detrimental effects of Cd^42^. Moreover, *Paenibacillus*, *Penicillium* and *Cladosporium* are known to directly absorb and immobilize soil Cd by exopolysaccharide production, formation of stable phosphate precipitates and oxidation of Mn (II) to biogenic manganese oxides (BMOs), respectively^43–45^. Thus, these rhizosphere microorganisms with diverse functionalities can synergistically enhance plant Cd tolerance through various mechanisms, including nutrient provision, gene expression regulation, chemical transformation, and direct Cd absorption/immobilization. They formed a complex functional network of rhizosphere microorganisms that promote healthy plant growth under Cd toxicity.

Microbial community composition and metabolite profiles exhibited significant variations among soybean varieties. In particular, the abundances of *Paenibacillus*, *Tumebacillus*, *Methylophilus*, *Aspergillus*, *Penicillium* and *Talaromyces* were positively correlated with sinapyl alcohol and coumestrol levels. Sinapyl alcohol serves as a precursor of coumestrol, a type of coumarin. Coumarin exudation, regulated by *MYB72*, have been shown to shape the root microbiome, promoting the health of *Arabidopsis thaliana*^18^. Therefore, our findings suggest that *GmAMT2.1/2.2* may enhance soybean adaptation to Cd toxicity by modulating the root microbiome through coumarin synthesis. *GmAMT2.1/2.2* could influence soil N content and alter the synthesis of flavonoids, including genistein, daidzin and daidzein (Fig. 6). These flavonoids may influence the symbiotic relationship between rhizobia and legumes, enriching beneficial microorganisms such as *Cladosporium* and *Aspergillus*, and further modulate plant defense responses^46,47^. In addition, low concentrations of the flavonoid chrysin can promote spore germination and root colonization by arbuscular mycorrhizal fungi, enhancing the rhizobia-plant symbiosis and potentially increasing soybean tolerance to Cd^48^. Moreover, *Paenibacillus*, *Ralstonia* and *Penicillium* were significantly correlated with piceatannol, a compound known to enhance plant resistance to biotic and abiotic stresses. This indicates that piceatannol may also selectively recruit beneficial microbes. Although a direct link between piceatannol and specific microbes has not yet been established, this could be a direction for future research.

All three SynComs alleviated Cd toxicity, with fungal SynCom and cross-kindom SynCom exhibiting greater efficacy than the bacterial SynCom (Fig. 7). This suggests that *GmAMT2.1/2.2* genes-regulated microbiome plays a crucial role in enhancing soybean resistance Cd toxicity, and fungi may hold greater potential than bacteria in assisting soybeans to adapt to Cd toxicity. Fungi mitigation of Cd toxicity involves diverse mechanisms, including metal biosorption onto the cell wall, intracellular accumulation and sequestration, and deposition of metal compounds within and around the mycelium^49^. Additionally, unlike bacteria, fungi can form extensive mycelial networks, facilitating bidirectional nutrient exchange with plants^50^. Additionally, the fungal SynCom may promote soybean adaptation Cd toxicity through the induction of genes involved in flavonoid and glucosinolate biosynthesis and ascorbic acid metabolism in the soybean root, which was not observed with the bacterial SynCom. These substances contribute to Cd toxicity mitigation by enriching certain beneficial microorganisms, increasing sulfur availability and scavenging excess H_2_O_2_ from plant cells^51–53^. Of note, a recent study by Xie et al. ^54^ showed that microorganisms might regulate the immune system responsible for Cd resistance in host plants^54^. This aligns with our finding that the bacterial SynCom specifically enriched for toll-like receptor signalling pathway, which served as a sensor for pathogens and enhances the immune response of plants^55,56^.

Furthermore, DEGs significantly upregulated by SynComs were involved in plant defense signalling, abiotic stress resistance and plant hormone signalling. It has been reported that lipoxygenases 1 (LOX1) and ZAT10 were directly related to Cd toxicity response. When *Arabidopsis thaliana* was exposed to Cd toxicity, LOX1 was involved in Cd-induced signalling transduction and increased jasmonic acid (JA) biosynthesis^57,58^. Similarly, ZAT10, a member of the C_2_H_2_ zinc finger gene family, negatively regulated Cd uptake in *Arabidopsis thaliana* and enhanced Cd detoxification, and positively regulating heavy metal detoxification-related genes such as NAS1, IRT2 and MTP3^59^. RD22, containing the plant-specific BURP domain, was also highly up-regulated under SynCom application and was involved in several abiotic resistances, including drought, salt and osmotic stresses^60,61^. CYP93D1, belonging to the cytochrome P450 monooxygenases family, is involved in JA metabolism and can be activated by ZmCLA4 to affect lignin biosynthesis^62^. Cd toxicity stimulates lignin biosynthesis, and the upregulation of CYP93D1 increases lignin content, which enhances Cd adsorption and promotes plant growth^63,64^. Two other significantly up-regulated DEGs under SynComs application are related to plant defense mechanisms. UGT73B3 is involved in regulating redox status and ROS reactivity, while ERF9 serves as a negative regulator of DREB AND EAR MOTIF PROTEIN 1 (DEAR1)-dependent ethylene/JA-mediated plant defense mechanisms^65,66^. Furthermore, phenylpropanoid biosynthesis was the key enriched pathway under SynComs treatments, indicating that SynComs activate the phenylpropanoid biosynthetic pathway, synthesizing lignin from the accumulated phenolic compounds to overcome heavy metal-induced stress^67^. In summary, SymComs application enables the coordinated regulation of LOX1, ZAT10, RD22, CYP93D1, UGT73B3 and ERF9 expression, triggering an interconnected network of defense responses, plant hormone signalling pathways, lignin biosynthesis and heavy metal detoxification mechanisms to enhance soybean’s ability to mitigate Cd toxicity.

It is noteworthy that the soil metabolites in this study are not the same as those from root exudates. Although the study used a single soil type, theoretically, the majority of the differences in soil metabolites are likely due to soybean genotypes. However, we cannot ignore the potential for soil microorganisms to be influenced by root exudates under *GmAMT2.1/2.2* genetic regulation, which could lead to the up-regulation or down-regulation of extracellular metabolites. Several metabolites from root exudates have been shown to alter soil microbial community composition^68^, suggesting that changes in the soil metabolite profile may partly due to extracellular compounds readily released by microorganisms. Thus, future studies should dedicate greater effort to distinguishing the relative importance of specific root- or soil-derived metabolites in shaping rhizosphere microbial structures^20,69^. Moreover, advanced techniques such as microfluidic-based cultivation and diversification, should be applied to isolate additional uncultivated microbes, including fungi and bacteria^28^. The efficacy of SynComs in promoting Cd tolerance in other crops and under field agricultural conditions requires further validation.

In summary, our study reveals that Cd toxicity induces the *GmAMT2.1/2.2*, leading to the recruitment of beneficial microorganisms, such as *Tumebacillus*, *Alicyclobacillus*, *Methylophilus*, *Aspergillus*, *Talaromyces*, and *Penicillium*, by altering metabolites such as sinapyl alcohol, genistein, coumestrol, and piceatannol. SynComs composed of these microorganisms collectively affecting various molecular mechanisms, thereby enhancing plant resistance to Cd toxicity. These mechanisms include ascorbate and aldarate metabolism and glucosinolate biosynthesis pathways associated with fungal SynCom, and plant-pathogen interaction and mineral absorption pathways associated with cross-kingdom SynCom. Notably, cross-kingdom SynCom and fungal SynCom showed greater potential for synergistic effects. Overall, our study results draw attention to the crucial role of microorganisms, especially fungi within SynComs, in the molecular mechanism underlying Cd toxicity resistance in soybean. The advanced insights provided by our metabarcoding data, and subsequent validation by microbial culture profiling, substantially enhance our understanding of plant functional gene regulation and synergistic resistance mechanisms within the root microbiome. This study provides critical data and guidance for the identification of agriculturally important microbial communities as potential breeding targets, as well as the development of microbial formulations to enhance resistance to Cd toxicity in sustainable agriculture.

## Materials and Methods

### Soil and soybean materials

Experimental soils were collected at the surface layer at a depth of 10 cm from acidic soils in China in the summer of 2020. The sampling site was Guangzhou (113°35′N, 23°15′E), Guangdong Province, which is classified as Udic Agrosol, according to USDA soil taxonomy. The characteristics of the soil were: pH 5.3, porosity 40.2, Cd 0.2 mg/kg, organic matter 5.2 g/kg, NH_4_^+^-N 5.3 mg/kg, available phosphorus (P) 15.5 mg/kg, NO_3_^-^-N 24 mg/kg, and available potassium (K) 85.2 mg/kg.

To construct the CaMV35S-drived *GmAMT2.2*, the full-length CDS of *GmAMT2.2* was cloned into the pTF101-eGFP vector, and the resultant construct was introduced into *Agrobacterium tumefaciens* EHA101. To produce the *GmAMT2.1* and *GmAMT2.2* loss-of-function mutants, the gene-editing tool CRISPR-Cas9 was used to knockout *GmAMT2.1* and *GmAMT2.2*. CRISPR/Cas9-mediated gene editing was performed as previously reported^70^. Briefly, the two sgRNAs targeting the first exon of *GmAMT2.1* and *GmAMT2.2* were designed on CRISPR-P server (http://crispr.hzau.edu.cn/CRISPR/)^71^. The sgRNAs was sub-cloned into pGES201 plasmid, and the resultant construct was further introduced into *Agrobacterium tumefaciens* EHA105. *Agrobacterium*-mediated transformation as previously reported^70,72^, and the soybean variety Young was used as the transgene receptor.

### RNA extraction and quantitative real-time PCR (qRT-PCR)

Total RNA was extracted from soybean or *Arabidopsis thaliana* with a TRNzol Universal Kit (DP424, TIANGEN, Beijing, China). The cDNA was synthesized from total RNA using a PrimeScript RT Reagent Kit with gDNA Eraser (RR047A, Takara Bio, Japan) according to the manufacturer’s instructions. DNA fragment amplification was performed using KOD FX neo (TOYOBO (SHANGHAI) BIOTECH CO., Shanghai, China). qRT-PCR was conducted using TB Green Premix Ex Taq II (RR820, Takara Bio, Japan) with a CFX96 Real-Time System (Bio-Rad, Hercules, CA, USA). Data were normalized to the reference genes *GmActin3*. All analyses were performed with three biological replicates and three technical replicates. The results were analyzed using the 2^–ΔΔCt^ method. Student’s *t*-test implemented in Excel software (Excel 2016) was used to evaluate the statistical significance of the data. The primers for the markers were listed in Supplementary Table 7.

### Experimental design

A pot experiment was conducted at the College of Agriculture, South China Agricultural University, located in Guangzhou, China. A randomized complete block design was used for the experiment, with three soybean genotypes (with or without Cd), for a total of six treatments (3 soybean genotypes × 2 Cd treatments = 6 treatments). Soybean seeds of the three genotypes were sterilized with alcohol and then planted in separate pots. The pots were of the same size and contained approximately 2.5 kg of soil. In each pot, eight seeds of uniform size were initially sown and subsequently thinned to two on the 10th day. The soil moisture content was maintained at 80% of the field capacity throughout the experiment. For each treatment, there were six replicates (plots). The soybeans were grown under controlled greenhouse conditions (day temperature 28 ~ 32 °C, night temperature 16 ~ 20 °C).

### Sample collection and soil chemical analysis

Twenty days after soybean sowing, 36 samples were collected and subjected to amplicon sequencing. Briley, the roots were shaken gently to remove the soil adhering to the roots. The roots and attached soil, which consider the rhizosphere soil, were then transferred to 1x phosphate-buffered saline. Ten grams of rhizosphere soil were obtained, of which two grams were stored at -80°C for the microbial experiment and LC-MS analysis. The remaining samples were stored at 4 °C for subsequent microbial isolation and soil chemical characterization measurements.

### Amplicon sequencing and data analysis

Utilizing the Fast DNA SPIN Kit for Soil (MP Biomedicals, Santa Ana, CA), microbial DNA was extracted, and the bacterial 16 S rRNA gene was specifically targeted by amplifying the V4 region using primers 515 F/806R^73^. Meanwhile, amplification of the fungal ITS region was achieved by targeting the ITS1 region using primers ITS5/1737 F. Briefly, each PCR reaction contained 4 μL buffer, 2 μL dNTPs (2 mM), 1 μL of forward/reverse primer (10 μM), 10 ng of DNA and 10 μL ddH_2_O. The PCR was then performed on an ABI 7900 system according to the following program 94 °C for 45 s; 35 cycles of 95 °C for 15 s, 55 °C for 10 s, 72 °C for 10 s; and 50 for 15 min. The amplicons that were pooled in equimolar amounts were subjected to paired-end sequencing on the Illumina MiSeq platform (Shanghai Majorbio Bio-pharm Technology Co., Ltd). The bacterial 16 S rRNA gene and fungal ITS sequences were processed with QIIME (v1.9.1) and VSEARCH, starting from the raw FASTQ files. After quality filtering, the primers and low-quality sequences with scores < 20 and lengths < 200 bp were removed. The paired-end reads that correspond to bacterial 16 S rRNA genes and fungal ITS regions were merged into single files, respectively. The operational taxonomic units (OTUs) were classified at a 97% sequence similarity cutoff using CD-HIT^74^. Taxonomic assignment was performed using the SILVA (v138) and UNITE (v8.0) databases for bacteria and fungi, respectively. Mothur and QIIME (v1.91) were used, respectively, to calculate the alpha and beta diversity of microbial communities. Rarefaction of bacterial and fungal OTU tables resulted in 60,024 and 60,049 reads, respectively, for alpha diversity estimation.

### Metabolite measurement

Fifty mg of the rhizosphere soil sample were added to a solvent of acetonitrile, methanol, and water (volume ratio 2:2:1). The sample was then vortexed and sonicated three times in an ice-water bath. The supernatant was centrifuged and subjected to UHPLC-Q Exactive (QE) Orbitrap MS analysis. Quality control samples were generated by pooling the supernatant from all samples and detecting soil metabolites via Liquid Chromatography-Tandem Mass Spectrometry (LC-MS/MS) methodology (Shanghai Majorbio Bio-pharm Technology Co., Ltd). The OPLS model was used to identify metabolites with the greatest group-specific differences based on their variable importance prediction (VIP) scores, with a threshold of 1 (OPLS, VIP > 1).

### High-throughput cultivation of microbes and construction of synthetic communities

The rhizosphere bacteria and fungi were isolated using high-throughput culture according to Zhang et al., (2021)^20^ and Zhou et al., (2022)^28^ with minor modifications. Briefly, the rhizosphere suspensions were diluted to an optimal dilution such that 30% of the wells showed bacterial growth. Then, 100 μL of the diluted suspension was plated on the 96-well plates containing different culture media. For the bacteria, the media included LB medium, beef extract peptone and Trytone yeast extract glucose medium. For the fungi, the media included 1/4 strength RBM, 1/4 strength RBM, 1/10 strength PDA and 1/4 strength MEA^28^. In addition, 0.01 M CdCl_2_ was added to the media to obtain bacteria and fungi more resistant to Cd. After 7 days of inoculation, microbial identification was used to identify each well. Bacterial 16 S rDNA and fungal 18 S rDNA were amplified and sequenced at Sangon Biotech Co., Ltd (Shanghai, China). Sequence alignment was performed on the NCBI website, and 1 mL of 30% glycerol (v/v) at -80°C was used to preserve each microbial species.

We identified bacteria and fungi that were significantly enriched in the rhizosphere of the soybean carrying *GmAMT2.1/2.2* genes by analyzing the composition of the rhizosphere microbial community in different treatments. These strains were then considered as potential candidates for building SynComs. The OD_600_ of each bacterial fermentation broth was adjusted to 0.02 (approximately 10^7^ cells/ml). Fungal strains were propagated using shake flask fermentation in 1/10th strength PDB medium, and diluted to 10^6^ conidia/ml^28^. To identify how root-derived microbes enhance plant adaptation to Cd toxicity, we conducted an experiment consisting of three synthetic SynComs, including bacterial, fungal, and bacterial and fungal cross-kingdom SynComs. Each SynCom consisted of an equal proportion of the respective bacterial or fungal strains. Different SynComs were added to the soil 10 days after soybean sowing. Plant fresh weight, root Cd^2+^ and NH_4_^+^-N content and translocation factor were determined 15 days after the addition of the SynComs.

### RNA‐seq for soybean treat with SynComs

RNA-seq was used to investigate the effect of SynComs on the expression of related genes in soybean. Total RNA was extracted from soybean root samples using RNAiso Plus reagent (Takara Bio). The constructed libraries underwent a thorough quality assessment before sequencing on the Illumina platform (PE 150). Then, the raw data files were converted into raw reads via base calling analysis. RSEM and STAR were used to perform the sequence the alignment and quantification of the detected genes^75^. Using the “stats” package in R 4.1.2, PCA (Principal components analysis) was conducted^76^ to show the effect of three SynComs on the gene expression of the soybean root. DESeq2 with the FDR < 0.05 was used to identify the differentially expressed genes (DEGs)^77^. A hierarchical clustering heatmap was employed to identify DEGs, which were subsequently annotated and analyzed for enriched pathways using the “stats” package’s hclust function^78^. The identified genes were annotated according to SoyBase (https://www.soybase.org) and then KEGG (http://www.genome.jp/kegg/) analysis was performed using the software package “clusterProfiler” to find significantly enriched metabolic or signalling pathways with a threshold of FDR < 0.05^79,80^.

### Statistical analysis

Principal coordinate analysis (PCoA) was conducted in R 4.1.2 using the “vegan” package, based on Bray-Curtis dissimilarities. The PERMANOVA (Permutation Multivariate Analysis of Variance) and the Mantel test were also used to evaluate the significance of the PCoA results. A generalized linear model was run in R using the “edgeR” package to analyze the microbial and metabolite differences between each two treatments. The results of the generalized linear model were visualized in volcano plots^81^. Using the “vcd” package, the Kruskal-Wallis test was performed in R to calculate the enrichment of microbes, and was then visualized in ternary plots using “ggplot2” package^82^. Differences in chemical properties of soil and microbial relative abundance on the phylum level were evaluated using Genstat (version 13.0) with the two-way analysis of variance (ANOVA). Analysis of the microbiome and metabolome data integration was performed using M^2^IA^83^. Microbial co-occurrence networks with an average OTU abundance greater than 0.1% across samples were constructed and analyzed to determine network connectivity in the genotypes. With the ’Hmisc’ and ’igraph’ packages in the R environment, spearman coefficients between OTUs were determined, and correlations with r > 0.8 and *P* < 0.05 were included in the network^84^. The networks were explored and visualized using Gephi (v 0.8.2). The codes used for the study are accessible on GitHub (https://github.com/liantengxiang1988/GmAMT2.1-2.2-shape-rhizosphere-microbiome-mitigate-cadmium-toxicity).

### Reporting summary

Further information on research design is available in the Nature Research Reporting Summary linked to this article.

### Supplementary information

Supplementary Information

Reporting summary

## Data Availability

The datasets generated for this study can be found in the NCBI short-read archive under accession number PRJNA798114 and PRJNA798115 for the bacteria and fungi, respectively. The RNA-seq data were submitted to NCBI database with the SRA accession number PRJNA983065.

## Code availability

The analysis and visualization codes utilized in this study have been included in the supplementary information of the manuscript.

## References

[1] Aslam, M. M., Okal, E. J. & Waseem, M. Cadmium toxicity impacts plant growth and plant remediation strategies. *Plant Growth Regul.* **99**, 397–412 (2023). 10.1007/s10725-022-00917-7

[2] Tóth, G., Hermann, T., Da Silva, M. R. & Montanarella, L. Heavy metals in agricultural soils of the European Union with implications for food safety. *Environ. Int.* **88**, 299–309 (2016). 26851498 10.1016/j.envint.2015.12.017

[3] Nagajyoti, P. C., Lee, K. D. & Sreekanth, T. V. M. Heavy metals, occurrence and toxicity for plants: a review. *Environ. Chem. Lett.* **8**, 199–216 (2010). 10.1007/s10311-010-0297-8

[4] Singh, S., Singh, A., Bashri, G. & Prasad, S. M. Impact of Cd stress on cellular functioning and its amelioration by phytohormones: An overview on regulatory network. *Plant Growth Regul.* **80**, 253–263 (2016). 10.1007/s10725-016-0170-2

[5] Shojaei, S., Jafarpour, A., Shojaei, S., Gyasi-Agyei, Y. & Rodrigo-Comino, J. Heavy metal uptake by plants from wastewater of different pulp concentrations and contaminated soils. *J. Clean. Prod.* **296**, 126345 (2021). 10.1016/j.jclepro.2021.126345

[6] Chen, L., He, L., Wang, Q. & Sheng, X. Synergistic effects of plant growth-promoting Neorhizobium huautlense T1-17 and immobilizers on the growth and heavy metal accumulation of edible tissues of hot pepper. *J. Hazard. Mater.* **312**, 123–131 (2016). 27017398 10.1016/j.jhazmat.2016.03.042

[7] Etesami, H. Bacterial mediated alleviation of heavy metal stress and decreased accumulation of metals in plant tissues: Mechanisms and future prospects. *Ecotoxicol. Environ. Saf.* **147**, 175–191 (2018). 28843189 10.1016/j.ecoenv.2017.08.032

[8] Hacquard, S., Spaepen, S., Garrido-Oter, R. & Schulze-Lefert, P. Interplay Between Innate Immunity and the Plant Microbiota. *Annu. Rev. Phytopathol.* **55**, 565–589 (2017). 28645232 10.1146/annurev-phyto-080516-035623

[9] Han, H. et al. Inhibition of cadmium uptake by wheat with urease-producing bacteria combined with sheep manure under field conditions. *Chemosphere* **293**, 133534 (2022). 34999099 10.1016/j.chemosphere.2022.133534

[10] Rajkumar, M., Sandhya, S., Prasad, M. N. V. & Freitas, H. Perspectives of plant-associated microbes in heavy metal phytoremediation. *Biotechnol. Adv.* **30**, 1562–1574 (2012). 22580219 10.1016/j.biotechadv.2012.04.011

[11] Sharma, R. K. & Archana, G. Cadmium minimization in food crops by cadmium resistant plant growth promoting rhizobacteria. *Appl. Soil Ecol.* **107**, 66–78 (2016). 10.1016/j.apsoil.2016.05.009

[12] Pishchik, V. N. et al. Experimental and mathematical simulation of plant growth promoting rhizobacteria and plant interaction under cadmium stress. *Plant Soil* **243**, 173–186 (2002). 10.1023/A:1019941525758

[13] Han, H., Wu, X., Yao, L. & Chen, Z. Heavy metal-immobilizing bacteria combined with calcium polypeptides reduced the uptake of Cd in wheat and shifted the rhizosphere bacterial communities. *Environ. Pollut.* **267**, 115432 (2020). 32841909 10.1016/j.envpol.2020.115432

[14] Beckers, B. et al. Lignin engineering in field-grown poplar trees affects the endosphere bacterial microbiome. *Proc. Natl Acad. Sci.* **113**, 201523264 (2016). 10.1073/pnas.1523264113 PMC4776533 26755604

[15] Chaparro, J. M., Sheflin, A. M., Manter, D. K. & Vivanco, J. M. Manipulating the soil microbiome to increase soil health and plant fertility. *Biol. Fertil. Soils* **48**, 489–499 (2012). 10.1007/s00374-012-0691-4

[16] Rodriguez, P. A. et al. Systems Biology of Plant-Microbiome Interactions. *Mol. Plant* **12**, 804–821 (2019). 31128275 10.1016/j.molp.2019.05.006

[17] Rolfe, S. A., Griffiths, J. & Ton, J. Crying out for help with root exudates: adaptive mechanisms by which stressed plants assemble health-promoting soil microbiomes. *Curr. Opin. Microbiol.* **49**, 73–82 (2019). 31731229 10.1016/j.mib.2019.10.003

[18] Stringlis, I. et al. MYB72-dependent coumarin exudation shapes root microbiome assembly to promote plant health. *Proc. Natl Acad. Sci.* **115**, 201722335 (2018). 10.1073/pnas.1722335115 PMC5984513 29686086

[19] Khanna, K., Jamwal, V. L., Gandhi, S., Ohri, P. & Bhardwaj, R. Metal resistant PGPR lowered Cd uptake and expression of metal transporter genes with improved growth and photosynthetic pigments in Lycopersicon esculentum under metal toxicity. *Sci. Rep.* **9**, 5855 (2019). 30971817 10.1038/s41598-019-41899-3 PMC6458120

[20] Zhang, J. et al. NRT1.1B is associated with root microbiota composition and nitrogen use in field-grown rice. *Nat. Biotechnol.* **37**, 676–684 (2019). 31036930 10.1038/s41587-019-0104-4

[21] Lian, T. et al. Rice SST Variation Shapes the Rhizosphere Bacterial Community, Conferring Tolerance to Salt Stress through Regulating Soil Metabolites. *mSystems* **5**, e00721–20 (2020). 33234605 10.1128/mSystems.00721-20 PMC7687028

[22] Chiasson, D. et al. Soybean SAT1 (Symbiotic Ammonium Transporter 1) encodes a bHLH transcription factor involved in nodule growth and NH 4 + transport. *Proc. Natl Acad. Sci. USA* **111**, 4814–4819 (2014). 24707045 10.1073/pnas.1312801111 PMC3977234

[23] Konishi, N. & Ma, J. F. Three polarly localized ammonium transporter 1 members are cooperatively responsible for ammonium uptake in rice under low ammonium condition. *N. Phytologist* **232**, 1778–1792 (2021). 10.1111/nph.17679 34392543

[24] Bu, Y., Takano, T. & Liu, S. The role of ammonium transporter (AMT) against salt stress in plants. *Plant Signal. Behav.* **14**, 1–3 (2019). 10.1080/15592324.2019.1625696 PMC6619917 31169446

[25] Vazquez, A., Recalde, L., Cabrera, A., Groppa, M. & Benavides, M. P. Does nitrogen source influence cadmium distribution in Arabidopsis plants? *Ecotoxicol. Environ. Saf.* **191**, 110163 (2020). 31951900 10.1016/j.ecoenv.2020.110163

[26] Yang, Y. et al. Regulatory mechanisms of nitrogen (N) on cadmium (Cd) uptake and accumulation in plants: A review. *Sci. Total Environ.* **708**, 135186 (2020). 31810697 10.1016/j.scitotenv.2019.135186

[27] Zhang, L.-D. et al. Ammonium has stronger Cd detoxification ability than nitrate by reducing Cd influx and increasing Cd fixation in Solanum nigrum L. *J. Hazard. Mater.* **425**, 127947 (2022). 34896722 10.1016/j.jhazmat.2021.127947

[28] Zhou, X. et al. Cross-kingdom synthetic microbiota supports tomato suppression of Fusarium wilt disease. *Nat. Commun.* **13**, 7890 (2022). 36550095 10.1038/s41467-022-35452-6 PMC9780251

[29] Liu, L. et al. Transgenic soybean of GsMYB10 shapes rhizosphere microbes to promote resistance to aluminum (Al) toxicity. *J. Hazard. Mater.* **455**, 131621 (2023). 37187122 10.1016/j.jhazmat.2023.131621

[30] Zhang, J. et al. High-throughput cultivation and identification of bacteria from the plant root microbiota. *Nat. Protoc.* **16**, 988–1012 (2021). 33442053 10.1038/s41596-020-00444-7

[31] Zhang, W. et al. Mycelial network-mediated rhizobial dispersal enhances legume nodulation. *ISME J.* **14**, 1015–1029 (2020). 31974462 10.1038/s41396-020-0587-5 PMC7082348

[32] Mir, I. R., Rather, B. A., Masood, A., Anjum, N. A. & Khan, N. A. Nitrogen Sources Mitigate Cadmium Phytotoxicity Differentially by Modulating Cellular Buffers, N-assimilation, Non-protein Thiols, and Phytochelatins in Mustard (Brassica juncea L.). *J. Soil Sci. Plant Nutr.* **22**, 3847–3867 (2022). 10.1007/s42729-022-00935-4

[33] Mir, I. R., Rather, B. A., Sehar, Z., Masood, A. & Khan, N. A. Nitric oxide in co-ordination with nitrogen reverses cadmium-inhibited photosynthetic activity by interacting with ethylene synthesis, strengthening the antioxidant system, and nitrogen and sulfur assimilation in mustard (Brassica juncea L.). *Sci. Horticulturae* **314**, 111958 (2023). 10.1016/j.scienta.2023.111958

[34] Jílková, V., Sim, A., Thornton, B. & Paterson, E. Grass rather than legume species decreases soil organic matter decomposition with nutrient addition. *Soil Biol. Biochem.* **177**, 108936 (2022). 10.1016/j.soilbio.2022.108936

[35] Liu, B. et al. Microbial metabolic efficiency and community stability in high and low fertility soils following wheat residue addition. *Appl. Soil Ecol.* **159**, (2020).

[36] Zhang, G. et al. Soil microbial communities regulate the threshold effect of salinity stress on SOM decomposition in coastal salt marshes. *Fundamental Res.*10.1016/j.fmre.2023.02.024 (2023). 10.1016/j.fmre.2023.02.024 PMC11197625 38933010

[37] Liu, X., Li, Q., Li, Y., Guan, G. & Chen, S. Paenibacillus strains with nitrogen fixation and multiple beneficial properties for promoting plant growth. *PeerJ* **7**, e7445 (2019). 31579563 10.7717/peerj.7445 PMC6761918

[38] Salcher, M., Schäfle, D., Kaspar, M., Neuenschwander, S. & Ghai, R. Evolution in action: habitat transition from sediment to the pelagial leads to genome streamlining in Methylophilaceae. *ISME J.* **13**, 2764–2777 (2019). 31292537 10.1038/s41396-019-0471-3 PMC6794327

[39] Yamagiwa, Y. et al. Talaromyces wortmannii FS2 emits β-caryphyllene, which promotes plant growth and induces resistance. *J. General Plant Pathol.* **77**, (2011).

[40] Huang, J., Liu, C., Price, G. W., Li, Y. & Wang, Y. Identification of a novel heavy metal resistant Ralstonia strain and its growth response to cadmium exposure. *J. Hazard. Mater.* **416**, 125942 (2021). 34492869 10.1016/j.jhazmat.2021.125942

[41] Wang, X.-H., Wang, Q., Nie, Z.-W., He, L.-Y. & Sheng, X.-F. Ralstonia eutropha Q2-8 reduces wheat plant above-ground tissue cadmium and arsenic uptake and increases the expression of the plant root cell wall organization and biosynthesis-related proteins. *Environ. Pollut.* **242**, 1488–1499 (2018). 30144722 10.1016/j.envpol.2018.08.039

[42] Aziz, L. et al. Endophytic Aspergillus niger reprograms the physicochemical traits of tomato under cadmium and chromium stress. *Environ. Exp. Bot.* **186**, 104456 (2021). 10.1016/j.envexpbot.2021.104456

[43] Morillo-Pérez, J. A. et al. Biosorption of heavy metals by the exopolysaccharide produced by Paenibacillus jamilae. *World J. Microbiol. Biotechnol.* **24**, 2699–2704 (2008). 10.1007/s11274-008-9800-9

[44] Wang, M. et al. An efficient manganese-oxidizing fungus Cladosporium halotolerans strain XM01: Mn(II) oxidization and Cd adsorption behavior. *Chemosphere* **287**, 132026 (2022). 34461328 10.1016/j.chemosphere.2021.132026

[45] Zheng, Y. et al. Efficient bioimmobilization of cadmium contamination in phosphate mining wastelands by the phosphate solubilizing fungus Penicillium oxalicum ZP6. *Biochem. Eng. J.* **187**, 108667 (2022). 10.1016/j.bej.2022.108667

[46] Han, Q. et al. Variation in rhizosphere microbial communities and its association with the symbiotic efficiency of rhizobia in soybean. *ISME J.* **14**, 1–14 (2020). 32336748 10.1038/s41396-020-0648-9 PMC7367843

[47] Sohn, S. I. et al. Metabolic Engineering of Isoflavones: An Updated Overview. *Front. Plant Sci.* **12**, 670103 (2021). 34163508 10.3389/fpls.2021.670103 PMC8216759

[48] Lidoy, J. et al. Flavonoids promote Rhizophagus irregularis spore germination and tomato root colonization: A target for sustainable agriculture. *Front. Plant Sci.* **13**, 1094194 (2023). 36684723 10.3389/fpls.2022.1094194 PMC9849897

[49] Ignatova, L. et al. Characterization of cadmium-tolerant endophytic fungi isolated from soybean (Glycine max) and barley (Hordeum vulgare). *Heliyon* **7**, e08240 (2021). 34765771 10.1016/j.heliyon.2021.e08240 PMC8570957

[50] Gutiérrez-Núñez, M. S., Gavito, M. E., Ortiz-Salgado, D. & Larsen, J. Agronomic practices and mycorrhizal development and function in maize: Root fungal interactions may affect early nutrition and yield. *Rhizosphere* **22**, 100525 (2022). 10.1016/j.rhisph.2022.100525

[51] Durenne, B., Druart, P., Blondel, A. & Fauconnier, M.-L. How cadmium affects the fitness and the glucosinolate content of oilseed rape plantlets. *Environ. Exp. Bot.* **155**, 185–194 (2018). 10.1016/j.envexpbot.2018.06.008

[52] Kaya, C., Ashraf, M., Alyemeni, M. N. & Ahmad, P. The role of nitrate reductase in brassinosteroid-induced endogenous nitric oxide generation to improve cadmium stress tolerance of pepper plants by upregulating the ascorbate-glutathione cycle. *Ecotoxicol. Environ. Saf.* **196**, 110483 (2020). 32247238 10.1016/j.ecoenv.2020.110483

[53] Yu, P. et al. Plant flavones enrich rhizosphere Oxalobacteraceae to improve maize performance under nitrogen deprivation. *Nat. Plants* **7**, 481–499 (2021). 33833418 10.1038/s41477-021-00897-y

[54] Xie, M. et al. Cadmium stimulated cooperation between bacterial endophytes and plant intrinsic detoxification mechanism in Lonicera japonica thunb. *Chemosphere* **325**, 138411 (2023). 36931404 10.1016/j.chemosphere.2023.138411

[55] Baker, B., Zambryski, P., Staskawicz, B. & Dinesh-Kumar, S. P. Signaling in Plant-Microbe Interactions. *Science* **276**, 726–733 (1997). 9115193 10.1126/science.276.5313.726

[56] Liu, Y., Zhang, Y.-M., Tang, Y., Chen, J.-Q. & Shao, Z.-Q. The evolution of plant NLR immune receptors and downstream signal components. *Curr. Opin. Plant Biol.* **73**, 102363 (2023). 37094492 10.1016/j.pbi.2023.102363

[57] Keunen, E. et al. A mutant of the Arabidopsis thaliana LIPOXYGENASE1 gene shows altered signalling and oxidative stress related responses after cadmium exposure. *Plant Physiol. Biochem.* **63**, 272–280 (2013). 23314084 10.1016/j.plaphy.2012.12.005

[58] Poveda, J. AtCube: Performing pathogen-root infection tests on Arabidopsis thaliana in a completely controlled way. *Physiol. Mol. Plant Pathol.* **117**, 101780 (2022). 10.1016/j.pmpp.2021.101780

[59] Dang, F. et al. ZAT10 plays dual roles in cadmium uptake and detoxification in Arabidopsis. *Front Plant Sci.* **13**, 994100 (2022). 36110357 10.3389/fpls.2022.994100 PMC9468636

[60] Harshavardhan, V. T. et al. AtRD22 and AtUSPL1, Members of the Plant-Specific BURP Domain Family Involved in Arabidopsis thaliana Drought Tolerance. *PLOS ONE* **9**, e110065 (2014). 25333723 10.1371/journal.pone.0110065 PMC4198191

[61] Wang, H. et al. Expression of an apoplast-localized BURP-domain protein from soybean (GmRD22) enhances tolerance towards abiotic stress. *Plant, cell Environ.* **35**, 1932–1947 (2012). 22548236 10.1111/j.1365-3040.2012.02526.x

[62] Dou, D. et al. CLA4 regulates leaf angle through multiple hormone signaling pathways in maize. *J. Exp. Bot.* **72**, 1782–1794 (2021). 33270106 10.1093/jxb/eraa565

[63] Demirbas, A. Adsorption of lead and cadmium ions in aqueous solutions onto modified lignin from alkali glycerol delignication. *J. Hazard. Mater.* **109**, 221–226 (2004). 15177762 10.1016/j.jhazmat.2004.04.002

[64] Yang, Y.-J., Cheng, L.-M. & Liu, Z.-H. Rapid effect of cadmium on lignin biosynthesis in soybean roots. *Plant Sci.* **172**(3), 632–639 (2007). 10.1016/j.plantsci.2006.11.018

[65] Maruyama, Y. et al. The Arabidopsis transcriptional repressor ERF9 participates in resistance against necrotrophic fungi. *Plant Sci.* **213**, 79–87 (2013). 24157210 10.1016/j.plantsci.2013.08.008

[66] Simon, C. et al. The secondary metabolism glycosyltransferases UGT73B3 and UGT73B5 are components of redox status in resistance of Arabidopsis to Pseudomonas syringae pv. tomato. *Plant, cell Environ.* **37**, 1114–1129 (2013). 24131360 10.1111/pce.12221

[67] Tavsan, Z. & Kayali, H. Phenylpropanoid Pathway Response to Cadmium and Lead Stress in Phaselous vulgaris Roots and Leaves. *Asian J. Biotechnol. Bioresour. Technol.* **3**, 1–11 (2018). 10.9734/AJB2T/2018/40759

[68] Wang, L. et al. Multifaceted roles of flavonoids mediating plant-microbe interactions. *Microbiome* **10**, 233 (2022). 36527160 10.1186/s40168-022-01420-x PMC9756786

[69] Lewis, W., Tahon, G., Geesink, P., Sousa, D. & Ettema, T. Innovations to culturing the uncultured microbial majority. *Nat. Rev. Microbiol.*10.1038/s41579-020-00458-8 (2020). 10.1038/s41579-020-00458-8 33093661

[70] Cai, Z. et al. CRISPR/Cas9-mediated gene editing of GmJAGGED1 increased yield in the low-latitude soybean variety Huachun 6. *Plant Biotechnol. J.* **19**, 1898 (2021). 34289223 10.1111/pbi.13673 PMC8486244

[71] Palindromic Repeats CRISPR-associated, I. S. CRISPR-P: A Web Tool for Synthetic Single-Guide RNA Design of CRISPR-System in Plants. (2014). 10.1093/mp/ssu044 24719468

[72] Li, S. et al. Optimization of Agrobacterium-mediated transformation in soybean. *Front. plant Sci.* **8**, 246 (2017). 28286512 10.3389/fpls.2017.00246 PMC5323423

[73] Walters, W. et al. Improved Bacterial 16S rRNA Gene (V4 and V4-5) and Fungal Internal Transcribed Spacer Marker Gene Primers for Microbial Community Surveys. *mSystems* **1**, e00009–e00015 (2015). 27822518 10.1128/mSystems.00009-15 PMC5069754

[74] Fu, L., Niu, B., Zhu, Z., Wu, S. & Li, W. CD-HIT: accelerated for clustering the next-generation sequencing data. *Bioinformatics* **28**, 3150–3152 (2012). 23060610 10.1093/bioinformatics/bts565 PMC3516142

[75] Li, B. & Dewey, C. N. RSEM: accurate transcript quantification from RNA-Seq data with or without a reference genome. *BMC Bioinforma.* **12**, 1–16 (2011). 10.1186/1471-2105-12-323 PMC3163565 21816040

[76] Liu, Y. et al. Metabolome and transcriptome analyses of the flavonoid biosynthetic pathway for the efficient accumulation of anthocyanins and other flavonoids in a new duckweed variety (68-red). *J. Plant Physiol.* **275**, 153753 (2022). 35760019 10.1016/j.jplph.2022.153753

[77] Love, M. I., Huber, W. & Anders, S. Moderated estimation of fold change and dispersion for RNA-seq data with DESeq2. *Genome Biol.* **15**, 1–21 (2014). 10.1186/s13059-014-0550-8 PMC4302049 25516281

[78] Zheng, L. et al. Global transcriptome analysis reveals dynamic gene expression profiling and provides insights into biosynthesis of resveratrol and anthraquinones in a medicinal plant Polygonum cuspidatum. *Ind. Crops Products* **171**, 113919 (2021). 10.1016/j.indcrop.2021.113919

[79] Kanehisa, M., Goto, S., Kawashima, S., Okuno, Y. & Hattori, M. The KEGG resource for deciphering the genome. *Nucleic acids Res.* **32**, D277–D280 (2004). 14681412 10.1093/nar/gkh063 PMC308797

[80] Yu, G., Wang, L.-G., Han, Y. & He, Q.-Y. clusterProfiler: an R package for comparing biological themes among gene clusters. *Omics: a J. Integr. Biol.* **16**, 284–287 (2012). 10.1089/omi.2011.0118 PMC3339379 22455463

[81] Robinson, M., Mccarthy, D. & Smyth, G. edgeR: a Bioconductor package for differential expression analysis of digital gene expression data. *Bioinformatics* **26**, 139–140 (2010). 19910308 10.1093/bioinformatics/btp616 PMC2796818

[82] Li, Z. et al. A simplified synthetic community rescues Astragalus mongholicus from root rot disease by activating plant-induced systemic resistance. *Microbiome* **9**, 217 (2021). 34732249 10.1186/s40168-021-01169-9 PMC8567675

[83] Ni, Y. et al. M2IA: a Web Server for Microbiome and Metabolome Integrative Analysis. *Bioinforma. (Oxf., Engl.)* **36**, 3493–3498 (2020). 10.1093/bioinformatics/btaa188 32176258

[84] Shi, Q. et al. Rhizosphere soil fungal communities of aluminum-tolerant and-sensitive soybean genotypes respond differently to aluminum stress in an acid soil. *Front. Microbiol.* **11**, 1177 (2020). 32547532 10.3389/fmicb.2020.01177 PMC7270577

