# Supplementary material for: GmAMT2.1/2.2-dependent ammonium nitrogen and metabolites shape rhizosphere microbiome assembly to mitigate cadmium toxicity
Source: NPJ Biofilms Microbiomes. 2024 Jul 24;10:60. doi: 10.1038/s41522-024-00532-6 (PMC11266425; doi:10.1038/s41522-024-00532-6)
Supplement: Supplementary file 1 — Supplementary Information [file 41522_2024_532_MOESM1_ESM.pdf]

GmAMT2.1/2.2-dependent ammonium nitrogen and metabolites shapes rhizosphere microbiome assembly to mitigate cadmium toxicity

Zhandong Cai[1,2#], Taobing Yu[1#], Weiyi Tan[1#], Qianghua Zhou[1], Lingrui Liu[1], Hai Nian[1*], and Tengxiang Lian[1*]

1 The Key Laboratory of Plant Molecular Breeding of Guangdong Province, College of Agriculture, South China Agricultural University, Guangzhou, Guangdong, China

2 Guangdong Provincial Key Laboratory of Utilization and Conservation of Food and Medicinal Resources in Northern Region, Shaoguan University, Shaoguan 512000, China

# Zhandong Cai, Taobing Yu and Weyi Tan contributed equally to this work.

*Corresponding author1: Tengxiang Lian

Corresponding address: No.483 Wushan Road, Guangzhou, Guangdong, 510642, China.

Tel: +86 02085288024; Fax: +86 02085288024; E-mail address: liantx@scau.edu.cn

*Corresponding author2: Hai Nian

Corresponding address: No.483 Wushan Road, Guangzhou, Guangdong, 510642, China.

Tel: +86 02085288024; Fax: +86 02085288024; E-mail address: hnian@scau.edu.cn

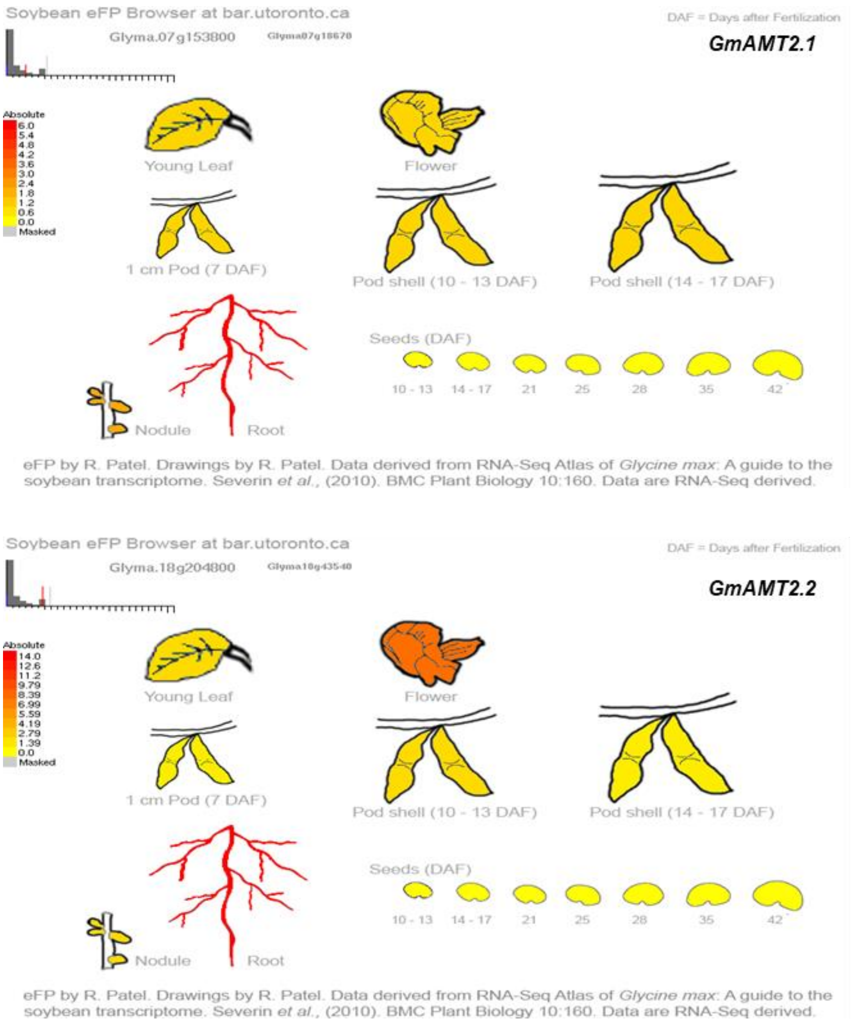

Supplementary Figure 1. Tissue expression patterns of *GmAMT2.1* (up) and *GmAMT2.2* (bottom). Data were retrieved from Soybean Expression Atlas (https://venanciogroup.uenf.br/cgi-bin/gmax_atlas/index.cgi).

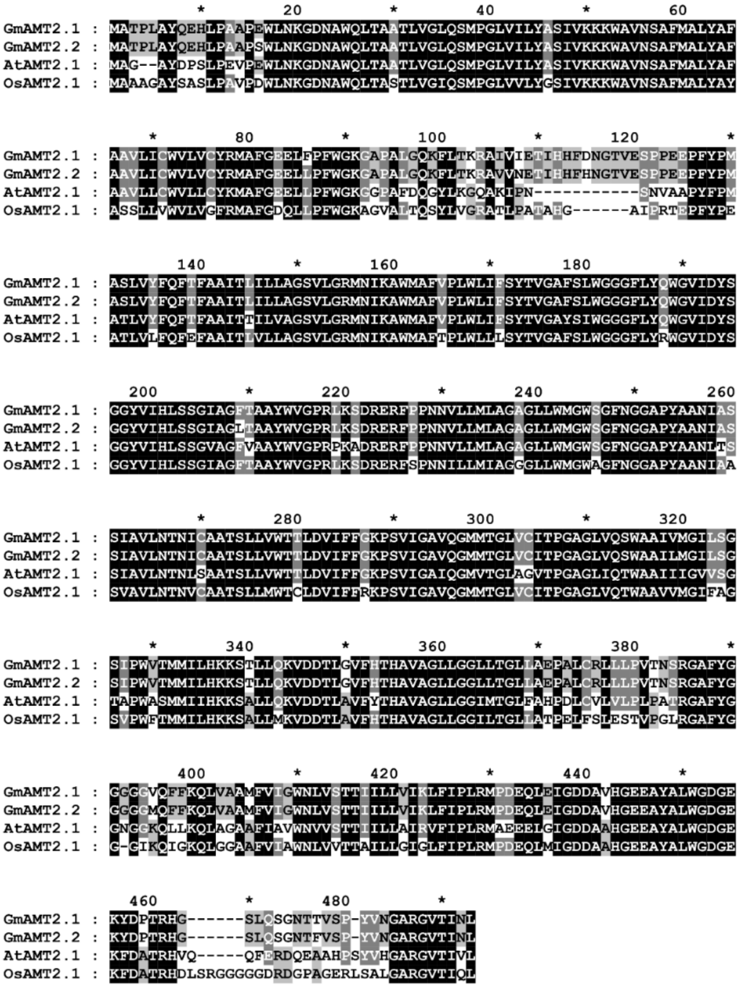

Supplementary Figure 2. Multiple sequence alignment of *GmAMT2.1*, *GmAMT2.2* and their homologues.

a

>GmAMT2.1
MATPLAYQEHLPAAPEWLNKGDNAWQLTAATLVGLQSMPGLVILYASIVKKK
WAVNSAFMALYAFAAVLICWVLVCYRMAFGEELFPFWGKGAPALGQKFLTKR
AIVIETIHHFDNGTVESPPEEPFYPMASLVYFQFTFAAITLILLAGSVLGRMNIKA
WMAFVPLWLIFSYTVGAFSLWGGGFLYQWGVIDYSGGYVIHLSSGIAGFTAAY
WVGPRLKSDRERFPPNNVLLMLAGAGLLWMGWSGFNGGAPYAANIASSIAVL
NTNICAATSLLVWTTLDVIFFGKPSVIGAVQGMMTGLVCITPGAGLVQSWAAIV
MGILSGSIPWVTMMILHKKSTLLQKVDDTLGVFHTHAVAGLLGGLLTGLLAEPA
LCRLLLPVTNSRGAFYGGGGGVQFFKQLVAAMFVIGWNLVSTTIILLVIKLFIPL
RMPDEQLEIGDDAVHGEEAYALWGDGEKYDPTRHGSLQSGNTTVSPYVNGA
RGVTINL*

>Mu1-Gmamt2.1(-5)
MATPLAYQEHLPAAPEWLNKGDNAWQLTAATLVGLQAGSRDPLRQHSEEKM
GSELSFHGLRLCGGSNMLGACVLPNGLWRRTFPLLGKGCSSTRPEVPHEKS
HSH*

>GmAMT2.2
MATPLAYQEHLPAAPSWLNKGDNAWQLTAATLVGLQSMPGLVILYASIVKKK
WAVNSAFMALYAFAAVLICWVLVCYRMAFGEELLPFWGKGAPALGQKFLTKR
AVVNETIHHFHNGTVESPPEEPFYPMASLVYFQFTFAAITLILLAGSVLGRMNIK
AWMAFVPLWLIFSYTVGAFSLWGGGFLYQWGVIDYSGGYVIHLSSGIAGLTAA
YWVGPRLKSDRERFPPNNVLLMLAGAGLLWMGWSGFNGGAPYAANIASSIAV
LNTNICAATSLLVWTTLDVIFFGKPSVIGAVQGMMTGLVCITPGAGLVQSWAAI
LMGILSGSIPWVTMMILHKKSTLLQKVDDTLGVFHTHAVAGLLGGLLTGLLAEP
ALCRLLLPVTNSRGAFYGGGGGMQFFKQLVAAMFVIGWNLVSTTIILLVIKLFIP
LRMPDEQLEIGDDAVHGEEAYALWGDGEKYDPTRHGSLQSGNTFVSPYVNG
ARGVTINL*

>Mu1-Gmamt2.2(-7)
MATPLAYQEHLPAAPSWLNKGDNAWQLTAATLVGLQSMPGLVILYASIVKKK
WAVNSAFMALYAFAAVLICWVLVCYRMAFGEELLPFWGKGAPALGQKFLTKR
AVVNETIHHFHNGTVESPPEEPFYPMASLVYFQFTFAAITLILLAGSVLGRMNIK
AWMAFVPLWLIFSYTVGAFSLWGGGFLYGLLTILAAMSSTFLLESLV*

b

>GmAMT2.1
MATPLAYQEHLPAAPEWLNKGDNAWQLTAATLVGLQSMPGLVILYASIVKKK
WAVNSAFMALYAFAAVLICWVLVCYRMAFGEELFPFWGKGAPALGQKFLTKR
AIVIETIHHFDNGTVESPPEEPFYPMASLVYFQFTFAAITLILLAGSVLGRMNIKA
WMAFVPLWLIFSYTVGAFSLWGGGFLYQWGVIDYSGGYVIHLSSGIAGFTAAY
WVGPRLKSDRERFPPNNVLLMLAGAGLLWMGWSGFNGGAPYAANIASSIAVL
NTNICAATSLLVWTTLDVIFFGKPSVIGAVQGMMTGLVCITPGAGLVQSWAAIV
MGILSGSIPWVTMMILHKKSTLLQKVDDTLGVFHTHAVAGLLGGLLTGLLAEPA
LCRLLLPVTNSRGAFYGGGGGVQFFKQLVAAMFVIGWNLVSTTIILLVIKLFIPL
RMPDEQLEIGDDAVHGEEAYALWGDGEKYDPTRHGSLQSGNTTVSPYVNGA
RGVTINL*

>Mu2-Gmamt2.1(+1)
MATPLAYQEHLPAAPEWLNKGDNAWQLTAATLVGLQSIAGSRDPLRQHSEEK
MGSELSFHGSLRLCGGSNMLGACVLPNGLWRRTFPLLGKGCSSTRPEVPHE
KSHSH*

>GmAMT2.2
MATPLAYQEHLPAAPSWLNKGDNAWQLTAATLVGLQSMPGLVILYASIVKKK
WAVNSAFMALYAFAAVLICWVLVCYRMAFGEELLPFWGKGAPALGQKFLTKR
AVVNETIHHFHNGTVESPPEEPFYPMASLVYFQFTFAAITLILLAGSVLGRMNIK
AWMAFVPLWLIFSYTVGAFSLWGGGFLYQWGVIDYSGGYVIHLSSGIAGLTAA
YWVGPRLKSDRERFPPNNVLLMLAGAGLLWMGWSGFNGGAPYAANIASSIAV
LNTNICAATSLLVWTTLDVIFFGKPSVIGAVQGMMTGLVCITPGAGLVQSWAAI
LMGILSGSIPWVTMMILHKKSTLLQKVDDTLGVFHTHAVAGLLGGLLTGLLAEP
ALCRLLLPVTNSRGAFYGGGGGMQFFKQLVAAMFVIGWNLVSTTIILLVIKLFIP
LRMPDEQLEIGDDAVHGEEAYALWGDGEKYDPTRHGSLQSGNTFVSPYVNG
ARGVTINL*

>Mu2-Gmamt2.2(+1)
MATPLAYQEHLPAAPSWLNKGDNAWQLTAATLVGLQSIAGSRDPLRKHSEEE
MGSEFSFHGSLCLCSSSNMLGACVLPHGLWRRTFTLLG*

Supplementary Figure 3. The mutation-caused amino acid alterations in Mu1 and Mu2. The * indicates the premature translation termination by the stop codon.

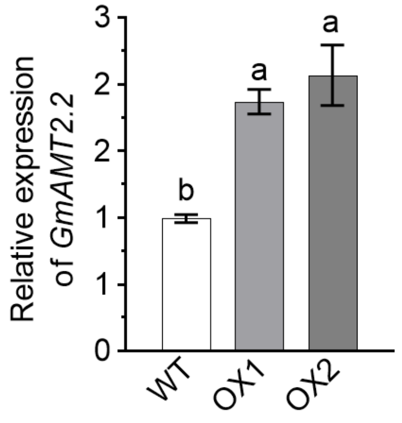

Supplementary Figure 4. Quantitative RT-PCR analysis of GmAMT2.2 in the overexpression lines.

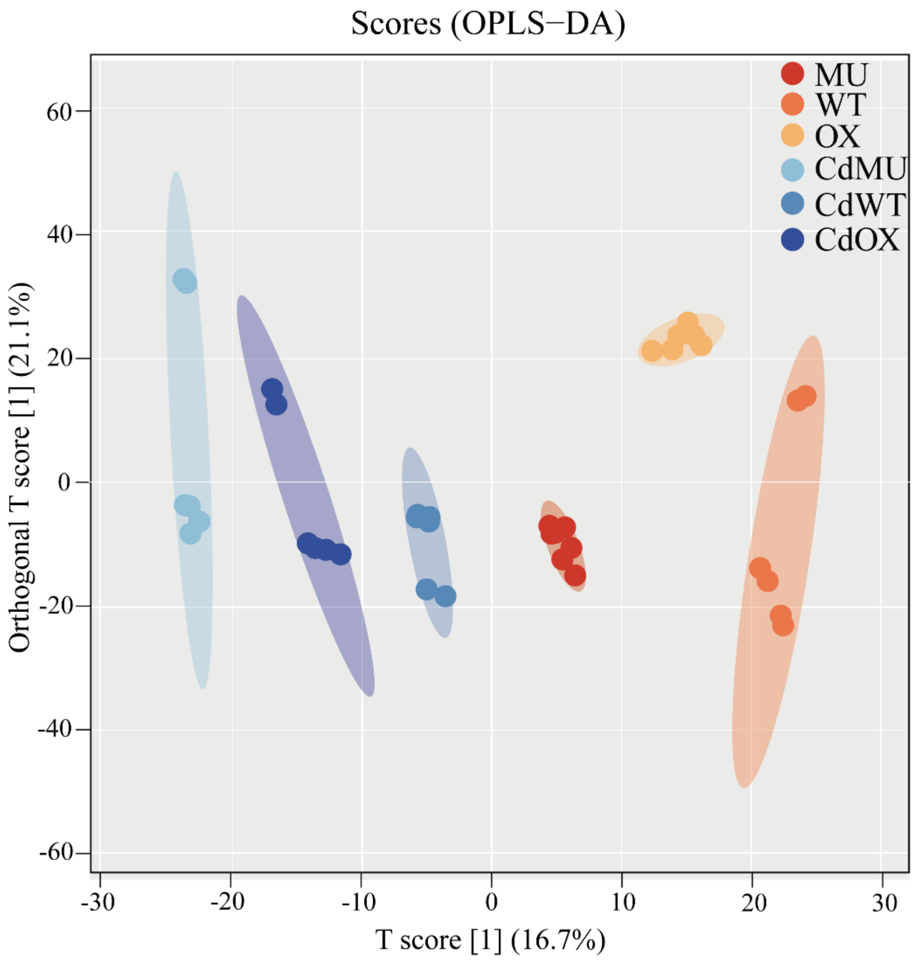

Supplementary Figure 5. Orthogonal partial least-squares discrimination analysis (OPLS-DA) (n = 6, $P < 0.05$) showing differences in soil metabolites between different treatment.

Supplementary Table 1. Effects of cadmium toxicity and soybean genotypes on microbial community structure assessed by permutational multivariate analysis of variance (PERMANOVA). Mu: *GmAMT2.1/2.2* double knockout lines; WT: wild type; OX: *GmAMT2.2* overexpression lines; Cd: cadmium toxicity.

Factor		F	R^2	*P*
Bacteria	Cd	9.1	0.19	0.001***
	Genotype	1.41	0.06	0.061
	Cd:Genotype	2.33	0.1	0.001***
Fungi	Cd	16.5	0.24	0.001***
	Genotype	4.43	0.13	0.001***
	Cd:Genotype	6.19	0.18	0.001***
**Pairwise comparison**		**F**	**R^2**	***P***
Bacteria	WT *vs.* OX	2.17	0.18	0.005**
	OX *vs.* MU	2.02	0.17	0.003**
	MU *vs.* WT	1.74	0.15	0.004**
	WT *vs.* CdWT	4.55	0.31	0.001**
	OX *vs.* CdOX	4.74	0.32	0.003**
	MU *vs.* CdMU	4.48	0.31	0.002**
	CdWT *vs.* CdOX	2.03	0.17	0.003**
	CdOX *vs.* CdMU	1.51	0.13	0.009**
Fungi	WT *vs.* OX	11.9	0.54	0.003**
	OX *vs.* MU	10.7	0.52	0.001***
	MU *vs.* WT	4.5	0.31	0.003**
	WT *vs.* CdWT	8.09	0.44	0.005**
	OX *vs.* CdOX	12.13	0.55	0.003**
	MU *vs.* CdMU	8.09	0.45	0.003**
	CdWT *vs.* CdOX	3.60	0.26	0.006**
	CdOX *vs.* CdMU	2.44	0.19	0.012*

Supplementary Table 2. Relative abundances (means ± SEs) of phyla showed a significant different response to cadmium toxicity and soybean genotypes as assessed by two way ANOVA. LSD: least significant difference. Mu: *GmAMT2.1/2.2* double knockout lines; WT: wild type; OX: *GmAMT2.2* overexpression lines; Cd: cadmium toxicity.

	Phylum	WT	OX	MU	CdWT	CdOX	CdMU	Cd	Genotype	Cd * Genotype	LSD
Bacteria	Proteobacteria	22.66±0.48	22.61±0.78	22.58±0.6	21.57±0.56	21.93±0.5	21.73±0.4	**<.001**	0.783	0.686	0.667
	Acidobacteriota	7.85±0.24	7.42±0.11	7.55±0.18	7.42±0.14	7.99±0.35	7.79±0.26	0.116	0.78	**<.001**	0.268
	Actinobacteriota	5.55±0.21	5.38±0.27	5.61±0.18	5.1±0.19	5.43±0.15	5.45±0.19	**0.008**	0.059	**0.015**	0.238
	Myxococcota	4.99±0.18	4.92±0.14	4.82±0.28	4.76±0.28	4.66±0.15	4.73±0.24	**0.014**	0.475	0.61	0.261
	Chloroflexi	5.3±0.36	5.15±0.16	5.18±0.19	5.4±0.9	5.03±0.17	4.99±0.09	0.613	0.209	0.655	0.488
	Firmicutes	3.7±0.15	3.74±0.09	3.77±0.18	3.51±0.08	3.53±0.09	3.57±0.1	**<.001**	0.429	0.979	0.141
	Verrucomicrobiota	4.17±0.46	3.39±0.27	3.7±0.13	3.59±0.09	3.78±0.19	3.51±0.29	0.179	**0.02**	**<.001**	0.316
	Gemmatimonadetes	2.29±0.11	2.34±0.03	2.28±0.11	2.41±0.18	2.4±0.12	2.5±0.14	**0.003**	0.779	0.306	0.146
	Other	43.49±0.81	45.05±0.89	44.51±0.58	46.24±0.98	45.26±0.76	45.75±0.68	**<.001**	0.615	**0.002**	0.937
Fungi	Ascomycota	68.97±1.72	61.52±5.92	70.27±2.58	62.09±2.32	66.72±2.35	63.71±4.31	**0.026**	0.153	**<.001**	4.147
	Basidiomycot	17.82±2.03	25.92±5.43	12.71±1.81	21.67±3.18	19.38±1.5	23.5±2.06	**0.011**	**0.003**	**<.001**	3.52
	Chytridiomycota	2.43±2.12	4.26±5.12	2.74±1.76	1.34±0.65	0.73±0.56	0.9±0.71	**0.012**	0.754	0.459	2.849
	Mortierellomycota	1.21±0.5	1.06±0.4	1.68±0.69	2±0.45	2.35±1.04	2.2±0.54	**<.001**	0.435	0.34	0.753
	Rozellomycota	0.47±0.23	0.39±0.19	1.09±0.71	0.8±0.63	0.86±0.34	1.53±1.55	0.119	0.057	0.973	0.902
	Glomeromycota	0.4±0.21	0.71±0.65	1.67±0.91	0.79±0.65	0.89±0.39	0.36±0.19	0.207	0.204	**0.001**	0.662
	Kickxellomycota	0.01±0.01	0.01±0.01	0.06±0.05	0.23±0.3	0.22±0.15	0.1±0.05	**0.002**	0.768	0.227	0.164
	Other	8.69±1.21	6.13±1.39	9.79±0.97	11.08±2.89	8.85±2	7.7±1.79	0.107	**0.011**	**0.004**	2.147

Supplementary Table 3. Topological characteristics of rhizosphere microbial networks across the different Cd toxicity and soybean genotypes. Mu: *GmAMT2.1/2.2* double knockout lines; WT: wild type; OX: *GmAMT2.2* overexpression lines; Cd: cadmium toxicity.

Topological characteristics	MU	WT	OX	CdMU	CdWT	CdOX
Number of nodes	192	197	190	196	190	202
Number of edges	528	578	510	663	597	581
Number of positive correlations	363	389	373	463	381	406
Number of negative correlations	165	189	136	199	209	190
Average path length (APL)	8.46	8.93	8.01	7.04	7.613	8.5
Graph density	0.03	0.03	0.023	0.035	0.033	0.029
Network diameter	22	24	19	22	21	21
Average clustering coefficient (avgCC)	0.69	0.71	0.69	0.72	0.71	0.69
Average degree (avgK)	5.5	5.86	5.36	6.76	6.22	5.91
Average weighted degree	2.15	1.98	2.49	2.896	1.833	2.13
Modularity (M)	0.74	0.74	0.71	0.61	0.68	0.74
Bacteria:Fungi	119:73	133:64	130:60	130:66	120:70	133:69

Supplementary Table 4. Effects of cadmium toxicity and soybean genotypes on soil metabolites assessed by permutational multivariate analysis of variance (PERMANOVA). Mu: *GmAMT2.1/2.2* double knockout lines; WT: wild type; OX: *GmAMT2.2* overexpression lines; Cd: cadmium toxicity.

Factor	F	R^2	*P*
Cd	22.1	0.29	0.001***
Genotype	6.75	0.18	0.001***
Cd:Genotype	4.64	0.12	0.001***
Pairwise comparison	F	R^2	*P*
WT *vs.* OX	16.8	0.63	0.002 **
OX *vs.* MU	63.4	0.87	0.005 **
MU *vs.* WT	12.2	0.55	0.005 **
WT *vs.* CdWT	9.24	0.48	0.004 **
OX *vs.* CdOX	19.1	0.66	0.005 **
MU *vs.* CdMU	12.9	0.56	0.003 **
CdWT *vs.* CdOX	2.09	0.17	0.155
CdOX *vs.* CdMU	3.51	0.26	0.02 *

Supplementary Table 5. VIP values of soil differential metabolites. WT: wild type; OX: *GmAMT2.2* overexpression lines; Cd: cadmium toxicity.

	ID	VIP	Log_2FC	*P*
CdWT vs WT （Depleted）	Sinapyl Alcohol	1.63	-1.22	<0.001
	Tussilagone	1.62	-1.13	<0.001
	Valeric acid	1.62	0.67	<0.001
	Acetyl-L-carnitine	1.62	-1.16	<0.001
	Toddalolactone	1.61	-1.23	<0.001
	Xanthosine Dihydrate	1.61	-1.22	<0.001
	L-Glutamic acid	1.61	-1.11	<0.001
	L-Glutamic acid	1.61	-1.11	<0.001
	Salicylic acid O-glucoside	1.61	-1.16	<0.001
	Sattabacin	1.61	-1.19	<0.001
	8,8'-dicarboxy-1,1'-binaphthalene	1.61	-1.14	<0.001
	Thymine	1.61	-1.10	<0.001
	5-O-Caffeoylshikimic acid	1.60	-1.17	<0.001
	L-Pyroglutamic acid	1.60	-1.11	<0.001
	Alisol B 23-acetate	1.60	-1.18	<0.001
	2'-Deoxyinosine	1.59	-1.19	<0.001
	4-Demethylepipodophyllotoxin	1.59	-1.12	<0.001
	Cytidine	1.59	-1.14	<0.001
	Uridine	1.59	-1.12	<0.001

	Pyrrole-2-carboxylic acid	1.59	-1.12	<0.001
	Aldosterone	1.59	-1.08	<0.001
	Fragransin A2	1.59	-1.09	<0.001
	Casticin	1.58	-1.16	<0.001
	Ethyl-氻-D-glucuronide	1.58	-1.23	<0.001
	5-Methyluridine	1.57	-1.10	<0.001
	Trehalose	1.57	-1.08	<0.001
	2'-Deoxyguanosine	1.46	-1.10	<0.001
	Guanine	1.41	-1.12	<0.001
	Xanthurenic acid	1.25	-1.11	<0.001
	Sucrose	1.16	-1.07	<0.012
CdOX vs OX（Enriched）	Genistein	1.92	1.22	<0.05
	Piceatannol	1.46	1.09	<0.001
	Chrysin	1.77	1.27	<0.001
	Daidzein	1.93	1.22	<0.05
	Daidzin	1.18	1.40	<0.05
	Glycitein	1.20	1.73	<0.05
	Coumestrol	1.21	1.85	<0.05
	4-methoxy-6-E-2-phenylethenyl-2H-pyran-2-one	1.13	1.63	<0.05
	Gomisin G	1.13	1.00	<0.001

Panaxydol	1.13	1.15	<0.001
Cinnamic Acid	1.12	1.08	<0.01
Caprylic acid	1.12	1.11	<0.01
LPC 141	1.12	1.02	<0.05
Sedanolide	1.12	1.12	<0.001
L-Phenylalanine	1.12	1.08	<0.01
L-Glutamine	1.12	1.06	<0.01
5-Methoxyindole-3-acetic acid	1.12	1.01	<0.01
2-4-aminophenoxyisophthalonitrile	1.12	1.15	<0.01
2-Phenylphenol	1.12	1.07	<0.01
3-hydroxy-1,5-diphenylpentan-1-one	1.12	1.11	<0.01
vasicine	1.12	1.21	<0.01
12Z-9,10,11-trihydroxyoctadec-12-enoic acid	1.12	1.23	<0.01
6E,10E-3,7,11,15-tetramethylhexadeca-1,6,10,14-tetraene-3,5,9-triol	1.12	1.02	<0.01
1-O-Palmitoylhexitol	1.12	1.13	<0.001
N-p-Coumaroylagmatine	1.12	1.07	<0.001
Ferruginol	1.12	1.04	<0.05
Linderane	1.11	1.09	<0.001
N', N''-p-Coumaroyl, Feruloylspermidine	1.11	1.06	<0.05
Isohomovanillic acid	1.11	1.20	<0.001

Psoralen	1.11	1.59	<0.01
Artesunate	1.11	1.08	<0.01
Quinone	1.11	1.12	<0.01
Kanosamine	1.11	1.23	<0.001
Tsugaric acid A	1.11	1.05	<0.05
2-Phenylacetamide	1.10	1.12	<0.01

Supplementary Table 6. Information of microbial strains in the three constructed SynComs.

		Microbial Strain	Strain assignment base on 16S RNA sequence and ITS sequencing.
SynCom3	SynCom1	*Paenibacillus doosanensis*	Bacteria;Firmicutes;Bacilli;Bacillales;Paenibacillaceae;Paenibacillus
		Paenibacillus cineris	Bacteria;Firmicutes; Bacilli; Paenibacillales; Paenibacillaceae; Paenibacillus
		Burkholderia cepacia	Bacteria;Proteobacteria;Betaproteobacteria;Burkholderiales;Burkholderiaceae;Burkholderia
		Burkholderia multivorans	Bacteria;Proteobacteria;Betaproteobacteria;Burkholderiales;Burkholderiaceae;Burkholderia
		Burkholderia seminalis	Bacteria;Proteobacteria;Betaproteobacteria;Burkholderiales;Burkholderiaceae;Burkholderia
		Burkholderia ubonensis	Bacteria;Proteobacteria;Betaproteobacteria;Burkholderiales;Burkholderiaceae;Burkholderia
	SynCom2	*Aspergillus fumigatus*	Fungi; Ascomycota; Pezizomycotina; Eurotiomycetes; Eurotiomycetidae; Eurotiales; Aspergillaceae; Aspergillus
		Aspergillus hiratsukae	Fungi; Ascomycota; Pezizomycotina; Eurotiomycetes; Eurotiomycetidae; Eurotiales; Aspergillaceae; Aspergillus
		Cladosporium caricinum	Fungi; Ascomycota; Pezizomycotina; Dothideomycetes; Dothideomycetidae; Cladosporiales; Cladosporiaceae; Cladosporium
		Cladosporium tenuissimum	Fungi; Ascomycota; Pezizomycotina; Dothideomycetes; Dothideomycetidae; Cladosporiales; Cladosporiaceae; Cladosporium
		Penicillium thomii	Fungi; Ascomycota; Pezizomycotina; Eurotiomycetes; Eurotiomycetidae; Eurotiales; Aspergillaceae; Penicillium
		Penicillium raperi	Fungi; Ascomycota; Pezizomycotina; Eurotiomycetes; Eurotiomycetidae; Eurotiales; Aspergillaceae; Penicillium
		Talaromyces funiculosus	Fungi; Ascomycota; Pezizomycotina; Eurotiomycetes; Eurotiomycetidae; Eurotiales; Trichocomaceae; Talaromyces;
		Papiliotrema laurentii	Fungi; Basidiomycota; Agaricomycotina; Tremellomycetes; Tremellales; Rhynchogastremataceae; Papiliotrema
		Papiliotrema laurentii	Fungi; Basidiomycota; Agaricomycotina; Tremellomycetes; Tremellales; Rhynchogastremataceae; Papiliotrema
		Talaromyces pinophilus	Fungi; Ascomycota; Pezizomycotina; Eurotiomycetes; Eurotiomycetidae; Eurotiales; Trichocomaceae; Talaromyces

Supplementary Table 7. Primers used in this study.

Primer name	Sequence (5' to 3')
CloneGmAMTF	ATGGCCACACCCTTGGCC
CloneGmAMTR	CATAAGTTTATGGTCACCC
Crispr/Cas9-T1F	GGATTGTTGGTCTTCAAAGCATGCC
Crispr/Cas9-T1R	AAACGGCATGCTTTGAAGACCAACA
Crispr/Cas9-T2F	GGATTGGTGGCTTTCTCTACCAATGG
Crispr/Cas9-T2R	AAACCCATTGGTAGAGAAAGCCACCA
pTF101-AMT-F	gagaacacgggggactctagaATGGCCACACCCTTGGCCTAC
pTF101-AMT -R	cgatcggggaaattcgagctcTCATAAGTTTATGGTCACCCCT
Actin3F	AAATTTCGGCATTGGCACCC
Actin3R	CACATCTGCTGGAAGGTGCT
qRT-GmAMT2.1-F	GTTGAATCACCTCCTGAGGAAC
qRT-GmAMT2.1-R	CCCGACTGTGTAGGAAAAGATC
qRT-GmAMT2.2-F	TGTTGGCTGGCTCTGTCCTCGGC
qRT-GmAMT2.2-R	TCCAGAAGAAAGGTGGATGAC

Codes used in this study

#PCoA

```
library(ggplot2)
library(vegan)
library(ape)
library(plyr)
df<-read.csv("otu.csv",row.names = 1)
aa<-df[,c(1:36)]
Treat<-
data.frame(c(rep(c("Mu"),6),rep(c("WT"),6),rep(c("OX"),6),rep(c("CdMu"),6),rep(c("CdWT"),6),
rep(c("CdOX"),6)))
Group<-data.frame(c(rep(c("CK"),18),rep(c("Cd"),18)))
data <- vegdist(t(aa), method = "bray")
pcoa<- pcoa(data, correction = "none", rn = NULL)
PCA1 = pcoa$vectors[,1]
PCA2 = pcoa$vectors[,2]
index<- data.frame(rownames(pcoa$vectors),PCA1,PCA2,Group,Treat)
colnames(index) <-c("sample","PCA1","PCA2","Group","Treat")
pca1 <-floor(pcoa$values$Relative_eig[1]*100)
pca2 <-floor(pcoa$values$Relative_eig[2]*100)
ggplot(index, aes(PCA1, PCA2,shape=Group))+ geom_point(aes(color=Treat),size=4)+
  xlab(paste("PCoA1 ( ",pca1,"%"," )",sep=""))+
  ylab(paste("PCoA2 ( ",pca2,"%"," )",sep=""))+
  #stat_ellipse(linetype="longdash",size=0.9,color="gray32")+
  #geom_polygon(data = index,aes(fill =Treat),alpha = 0.5,show.legend = FALSE) +

scale_color_manual(values=c("#1373B2","#EF8A47","#F7AA58","#FFD06F","#FFE6B7","#E76
254"))+
scale_fill_manual(values=c("#1373B2","#EF8A47","#F7AA58","#FFD06F","#FFE6B7","#E7625
4"))+ theme_bw()+
  theme(axis.text=element_text(colour='black',size=18))+
  theme(panel.grid.major=element_blank(),panel.grid.minor=element_blank())
```

#Volcano plot

```
library(ggplot2)
data <- read.csv("1111.csv",row.names = 1)
head(data)
data$label <- c(rownames(data)[1:10],rep(NA,(nrow(data)-10)))
ggplot(data,aes(log2FoldChange, -log10(padj)))+
  geom_hline(yintercept = -log10(0.05), linetype = "dashed", color = "#999999")+
  geom_vline(xintercept = c(-1.2,1.2), linetype = "dashed", color = "#999999")+
  geom_point(aes(size=-log10(padj), color= -log10(padj)))+
```

```
    scale_color_gradientn(values = seq(0,1,0.2),
                                            colors = c("#39489f","#39bbec","#f9ed36","#f38466","#b81f25"))+
    scale_size_continuous(range = c(1,3))+
    theme_bw()+
    theme(panel.grid = element_blank(),
              legend.position = c(0.01,0.7),
              legend.justification = c(0,1)
    )+
    guides(col = guide_colourbar(title = "-Log10_q-value"),
               size = "none")+
    geom_text(aes(label=label, color = -log10(padj)), size = 3, vjust = 1.5, hjust=1)+
    xlab("Log2FC")+
    ylab("-Log10(FDR q-value)")
```

#Box plot

```
library(ggplot2)
library(RColorBrewer)
library(ggsci)
library(ggpubr)
aa<-read.csv("J://test/test_otu.csv",row.names = 1)
df<-aa[21:220,1:3]
#df<- as.data.frame(scale(df))
Group<-data.frame(c(rep(c(4:8,4:8,4:8,4:8),10)),c(rep(c(1),50)))
colnames(Group)<-c("Shannon","Group")
df<-cbind(df,Group)
df$pH<-as.factor(df$Shannon)
df$Sample3<-"Box plot"
p1=ggplot(df, aes(x=Shannon, y=Sample2,color=Shannon))+
    stat_boxplot(geom="errorbar",position=position_dodge(width=0.2),width=0.3,size=1)+
    geom_boxplot(aes(),notch = F,size=0.7)+
    geom_jitter(size = 2.5,alpha = 0.6,width = 0.2)+
    theme_bw()+scale_color_npg()+scale_fill_npg()+
    theme(axis.text=element_text(colour='black',size=9))+
    labs(x="Metabolic", y="Growth", color = "Shannon",fill = "Shannon")+
    facet_grid( ~Sample3, drop=TRUE,scale="free", space="free_x")+
    theme(strip.background = element_rect(fill=c("white")))+
    theme(strip.text = element_text(size = 12,face = 'bold',colour = "gray2"))+
    theme(axis.text=element_text(colour='black',size=11))
plot_data2<-df
mean_df<-aggregate(plot_data2[,1:2],by=list(plot_data2[,4]),FUN=mean)
rownames(mean_df)<-mean_df[,1]
sd_df<-aggregate(plot_data2[,1:2],by=list(plot_data2[,4]),FUN=sd)
rownames(sd_df)<-sd_df[,1]
sd_df<-sd_df[,-1]
```

```
se_df<-sd_df/sqrt(40) # n=40
se<-as.data.frame(se_df)
plot_data1<-cbind(mean_df,se)
colnames(plot_data1)<-c("Shannon","Sample1","Sample2","se1","se2")
#barplot
df$Sample3<-"Bar plot"
#barplot
p2=ggplot(df, aes(x=Shannon, y=Sample1,color=Shannon))+
  geom_bar(data=plot_data1,mapping=aes(x=Shannon,y=Sample1,fill=Shannon), size =
1.03,position="dodge", stat="identity",width = 0.7)+
  geom_errorbar(data=plot_data1,mapping=aes(x=Shannon,ymin=Sample1-se1,
ymax=Sample1+se1),width = 0.3,size=0.8)+
  geom_jitter(size = 2.5,alpha = 0.6,width = 0.2)+
  theme_bw()+scale_color_npg()+scale_fill_npg()+
  theme(axis.text=element_text(colour='black',size=9))+
  labs(x="Metabolic", y="Growth", color = "Shannon",fill = "Shannon")+
  facet_grid( ~Sample3, drop=TRUE,scale="free", space="free_x")+
  theme(strip.background = element_rect(fill=c("white")))+
  theme(strip.text = element_text(size = 12,face = 'bold',colour = "gray2"))+
  theme(axis.text=element_text(colour='black',size=11))
```

#network

```
library(Hmisc)
otu=read.table("otu.txt", head=T, row.names=1)
occor <- rcorr(as.matrix(otu),type = 'spearman')
occor.r <-   occor$r
occor.p <-   occor$P
occor.p<-p.adjust(occor.p, method="BH")
occor.r[occor.p<0.01|abs(occor.r)<0.8] = 0
diag(occor.r) <- 0
write.csv(occor.r,file="NOC.csv")
```

#PCA

```
rm(list = ls())
data0 = read.table("S.txt", header=TRUE,sep='\t',row.names=1)
library(ggplot2)
library(ggrepel)
#devtools::install_github("vqv/ggbiplot")
library(ggbiplot)
library(RColorBrewer)
library(grid)
cols <- c("Heat-killed" = "grey", "SynCom1" = "#e64b35", "SynCom2" = "#3c5488", "SynCom3"
= "#91d1c2")
mydata1 = t(as.matrix(data0))
```

```
mydata.pca <- prcomp(log(mydata1+1))
scores <- mydata.pca$x
scores=data.frame(scores)
rownames(scores)=colnames(data0)
scores$Subtype = c("Heat-
killed",'SynCom3',rep("SynCom1",33),"SynCom2","SynCom2","SynCom1",rep("Heat-
killed",3),'SynCom2',rep("Heat-killed",2),rep("SynCom1",3),'SynCom2')
N1=as.character(round(summary(mydata.pca)$importance[3,1],2))
N2=as.character(round(summary(mydata.pca)$importance[3,2]-
summary(mydata.pca)$importance[3,1],2))
N3=as.character(round(summary(mydata.pca)$importance[3,3]-
summary(mydata.pca)$importance[3,2],2))
xl=paste("PC1 ","(",N1,")",sep='')
yl=paste("PC2 ","(",N2,")",sep='')
zl=paste("PC3 ","(",N3,")",sep='')
mytheme<-theme_test()+
  theme(panel.background = element_rect(fill = "white", colour = "black"),
        panel.grid.major = element_blank(),panel.grid.minor = element_blank(),
        plot.title = element_text(hjust = 0.5,size = 15),
        axis.text = element_text(size = 8),axis.title = element_text(size = 12,face="bold"),
        legend.text = element_text(size = 12),legend.title = element_text(size = 15),
        plot.margin = unit(c(0.2,0.2,0.2,0.2),'cm'))
ggplot(scores, aes(PC1, PC2,colour = Subtype)) +
    geom_point(size=3.5) +
    xlab(xl) + ylab(yl)   + scale_colour_manual(values = cols) +
  mytheme+
  theme(legend.position = c(0.9,0.3),
        legend.key   = element_rect(fill = 'grey95'))
```

#Heatmap

```
library(openxlsx)
data<- read.xlsx("Genus.xlsx", sheet = 1, startRow = 1, colNames = TRUE, rowNames = TRUE,
detectDates = FALSE, skipEmptyRows = TRUE, skipEmptyCols = TRUE, rows = NULL, cols =
NULL, check.names = FALSE, sep.names = ".", namedRegion = NULL, na.strings = "NA",
fillMergedCells = FALSE)
data
# data<-cor(data,method = "spearman")
# Samplegroup= read.xlsx("D://test",header = T,row.names = 1,fill = T,sep = "\t")
library(pheatmap)
# ?pheatmap
pheatmap(data,scale = "none")
pheatmap(data,scale = "row")
pheatmap(data,scale = "column")
annotation_col=data.frame(Sample=factor(c(rep("M1",3),rep("M2",3),rep("M3",3),rep("M4",3))))
```

```
row.names(annotation_col) = colnames(data)
ann_colors = list(Sample = c(M1="#E889BD", M2="#B286D7", M3="#5189E0",
M4="#0089CF"))
pheatmap(data,
        scale = "row",
        cluster_cols = FALSE, clustering_distance_rows = "correlation",
        treeheight_row = 30,
        annotation_col = annotation_col,
        annotation_colors = ann_colors
```

#KEGG

```
install.packages("dplyr")
install.packages("ggplot2")
install.packages("tidyverse")
install.packages("openxlsx")
library (dplyr)
library (ggplot2)
library(tidyverse)
library(openxlsx)
kk_result= read.xlsx('C:/Rdata/jc/enrich-gene.xlsx', sheet= "KEGG", sep = ',')
display_number = 30
kk_result = as.data.frame(kk_result)[1:display_number[1], ]
kk = as.data.frame(kk_result)
rownames(kk) = 1:nrow(kk)
kk$order=factor(rev(as.integer(rownames(kk))),labels = rev(kk$Description))
ggplot(kk,aes(y=order,x=Gene_Ratio))+
    geom_point(aes(size=Count,color=PValue))+
    scale_color_gradient(low = "red",high ="blue")+
    labs(color=expression(PValue,size="Count"),
      x="Gene Ratio",y="Pathways",title="KEGG Pathway Enrichment")+
  theme_bw()
```
