# Supplementary material for: GmAMT2.1/2.2-dependent ammonium nitrogen and metabolites shape rhizosphere microbiome assembly to mitigate cadmium toxicity
Source: NPJ Biofilms Microbiomes. 2024 Jul 24;10:60. doi: 10.1038/s41522-024-00532-6 (PMC11266425; doi:10.1038/s41522-024-00532-6)
Supplement: Supplementary file 2 — Reporting summary [file 41522_2024_532_MOESM2_ESM.pdf]

nature portfolio

Corresponding author(s): *Double-anonymous peer review submissions: write DAPR and your manuscript number here instead of author names.*

Last updated by author(s): *YYYY-MM-DD*

Reporting Summary

Nature Portfolio wishes to improve the reproducibility of the work that we publish. This form provides structure for consistency and transparency in reporting. For further information on Nature Portfolio policies, see our Editorial Policies and the Editorial Policy Checklist.

Statistics

For all statistical analyses, confirm that the following items are present in the figure legend, table legend, main text, or Methods section.

n/a	Confirmed	
☐	☒	The exact sample size (*n*) for each experimental group/condition, given as a discrete number and unit of measurement
☐	☒	A statement on whether measurements were taken from distinct samples or whether the same sample was measured repeatedly
☐	☒	The statistical test(s) used AND whether they are one- or two-sided<br>*Only common tests should be described solely by name; describe more complex techniques in the Methods section.*
☐	☒	A description of all covariates tested
☐	☒	A description of any assumptions or corrections, such as tests of normality and adjustment for multiple comparisons
☐	☒	A full description of the statistical parameters including central tendency (e.g. means) or other basic estimates (e.g. regression coefficient) AND variation (e.g. standard deviation) or associated estimates of uncertainty (e.g. confidence intervals)
☐	☒	For null hypothesis testing, the test statistic (e.g. *F*, *t*, *r*) with confidence intervals, effect sizes, degrees of freedom and *P* value noted<br>*Give P values as exact values whenever suitable.*
☐	☒	For Bayesian analysis, information on the choice of priors and Markov chain Monte Carlo settings
☐	☒	For hierarchical and complex designs, identification of the appropriate level for tests and full reporting of outcomes
☐	☒	Estimates of effect sizes (e.g. Cohen's *d*, Pearson's *r*), indicating how they were calculated

Our web collection on statistics for biologists contains articles on many of the points above.

Software and code

Policy information about availability of computer code

Data collection	The codes used for the study are accessible on GitHub (https://github.com/liantengxiang1988/GmAMT2.1-2.2-shape-rhizosphere-microbiome-mitigate-cadmium-toxicity).
Data analysis	Principal coordinate analysis (PCoA) was conducted in R 4.1.2 using the "vegan" package, based on Bray-Curtis dissimilarities. The PERMANOVA (Permutation Multivariate Analysis of Variance) and the Mantel test were also used to evaluate the significance of the PCoA results. A generalized linear model was run in R using the "edgeR" package to analyze the microbial and metabolite differences between each two treatments. The results of the generalized linear model were visualized in volcano plots44. Using the “vcd” package, the Kruskal-Wallis test was performed in R to calculate the enrichment of microbes, and was then visualized in ternary plots using “ggplot2” package45. Differences in chemical properties of soil and microbial relative abundance on the phylum level were evaluated using Genstat (version 13.0) with the two-way analysis of variance (ANOVA). Analysis of the microbiome and metabolome data integration was performed using M2IA46. Microbial co-occurrence networks with an average OTU abundance greater than 0.1% across samples were constructed and analyzed to determine network connectivity in the genotypes. With the ’Hmisc’ and ’igraph’ packages in the R environment, spearman coefficients between OTUs were determined, and correlations with r > 0.8 and P < 0.05 were included in the network47. The networks were explored and visualized using Gephi (v 0.8.2).

For manuscripts utilizing custom algorithms or software that are central to the research but not yet described in published literature, software must be made available to editors and reviewers. We strongly encourage code deposition in a community repository (e.g. GitHub). See the Nature Portfolio guidelines for submitting code & software for further information.

Data

Policy information about availability of data

All manuscripts must include a data availability statement. This statement should provide the following information, where applicable:

- Accession codes, unique identifiers, or web links for publicly available datasets
- A description of any restrictions on data availability
- For clinical datasets or third party data, please ensure that the statement adheres to our policy

The datasets generated for this study can be found in the NCBI short-read archive under accession number PRJNA798114 and PRJNA798115 for the bacteria and fungi, respectively. The RNA-seq data were submitted to NCBI database with the SRA accession number PRJNA983065.

Research involving human participants, their data, or biological material

Policy information about studies with human participants or human data. See also policy information about sex, gender (identity/presentation), and sexual orientation and race, ethnicity and racism.

Reporting on sex and gender	This study is not involving human participants.
Reporting on race, ethnicity, or other socially relevant groupings	This study is not involving human participants.
Population characteristics	This study is not involving human participants.
Recruitment	This study is not involving human participants.
Ethics oversight	This study is not involving human participants.

Note that full information on the approval of the study protocol must also be provided in the manuscript.

Field-specific reporting

Please select the one below that is the best fit for your research. If you are not sure, read the appropriate sections before making your selection.

☐ Life sciences ☐ Behavioural & social sciences ☒ Ecological, evolutionary & environmental sciences

For a reference copy of the document with all sections, see nature.com/documents/nr-reporting-summary-flat.pdf

Ecological, evolutionary & environmental sciences study design

All studies must disclose on these points even when the disclosure is negative.

Study description	A pot experiment was conducted at the College of Agriculture, South China Agricultural University, located in Guangzhou, China. A randomized complete block design was used for the experiment, with three soybean genotypes (with or without Cd), for a total of six treatments (3 soybean genotypes × 2 Cd treatments = 6 treatments).
Research sample	Twenty days after soybean sowing, 36 samples were collected and subjected to amplicon sequencing.
Sampling strategy	Briley, the roots were shaken gently to remove the soil adhering to the roots. The roots and attached soil, which consider the rhizosphere soil, were then transferred to 1x phosphate-buffered saline. Ten grams of rhizosphere soil were obtained, of which two grams were stored at -80°C for the microbial experiment and LC-MS analysis.
Data collection	The first authors used the Fast DNA SPIN Kit for Soil (MP Biomedicals, Santa Ana, CA) extracted microbial DNA, and the bacterial 16S rRNA gene was specifically targeted by amplifying the V4 region using primers 515F/806R35. Meanwhile, amplification of the fungal ITS region was achieved by targeting the ITS1 region using primers ITS5/1737F.
Timing and spatial scale	Twenty days after soybean sowing, 36 samples were collected and subjected to amplicon sequencing.
Data exclusions	no data were excluded
Reproducibility	all attempts to repeat the experiment were successful
Randomization	A randomized complete block design was used for the experiment, with three soybean genotypes (with or without Cd), for a total of six treatments (3 soybean genotypes × 2 Cd treatments = 6 treatments).
Blinding	The study investigated the effects of genetic modifications in soybeans on their rhizosphere microbiota and Cd resistance. Blinding is

Blinding	typically relevant for studies where participants or researchers are unaware of the intervention or treatment received by the subjects. In this study, the "intervention" is the genetic modification, which is inherent to the plants and cannot be disguised or blinded.

Did the study involve field work? ☐ Yes ☒ No

Reporting for specific materials, systems and methods

We require information from authors about some types of materials, experimental systems and methods used in many studies. Here, indicate whether each material, system or method listed is relevant to your study. If you are not sure if a list item applies to your research, read the appropriate section before selecting a response.

Materials & experimental systems

n/a	Involved in the study	
☒	☐	Antibodies
☒	☐	Eukaryotic cell lines
☒	☐	Palaeontology and archaeology
☒	☐	Animals and other organisms
☒	☐	Clinical data
☒	☐	Dual use research of concern
☐	☒	Plants

Methods

n/a	Involved in the study	
☒	☐	ChIP-seq
☒	☐	Flow cytometry
☒	☐	MRI-based neuroimaging

Dual use research of concern

Policy information about dual use research of concern

Hazards

Could the accidental, deliberate or reckless misuse of agents or technologies generated in the work, or the application of information presented in the manuscript, pose a threat to:

No	Yes	
☒	☐	Public health
☒	☐	National security
☐	☒	Crops and/or livestock
☒	☐	Ecosystems
☒	☐	Any other significant area

Hazards	there is no hazrds of this study.

For examples of agents subject to oversight, see the United States Government Policy for Institutional Oversight of Life Sciences Dual Use Research of Concern.

Experiments of concern

Does the work involve any of these experiments of concern:

No	Yes	
☒	☐	Demonstrate how to render a vaccine ineffective
☒	☐	Confer resistance to therapeutically useful antibiotics or antiviral agents
☒	☐	Enhance the virulence of a pathogen or render a nonpathogen virulent
☒	☐	Increase transmissibility of a pathogen
☒	☐	Alter the host range of a pathogen
☒	☐	Enable evasion of diagnostic/detection modalities
☒	☐	Enable the weaponization of a biological agent or toxin
☒	☐	Any other potentially harmful combination of experiments and agents

Precautions and benefits

Biosecurity precautions	The knowledge gained from the study could be used to develop genetically modified plants with increased resistance to toxic metals, potentially creating environmental hazards if released accidentally or intentionally.

Biosecurity oversight

The study of genetic modification of plants could raise potential biosecurity concerns. However, we have strict external oversight to ensure compliance with biosafety guidelines.

Benefits

Reduced reliance on chemical fertilizers: By enhancing nitrogen uptake, these genes could decrease the need for synthetic nitrogen fertilizers, which have significant environmental impacts through greenhouse gas emissions and water pollution.
Improved soil health: The rhizosphere microbial communities facilitated by GmAMT2.1/2.2 genes may enhance soil health by promoting nutrient cycling and organic matter decomposition.
Enhanced plant resilience to Cd stress: The research findings could contribute to developing Cd-resistant soybean varieties, reducing the risk of Cd uptake into the food chain and protecting human health.
Reduced land use for agriculture: Increased nitrogen use efficiency through GmAMT2.1/2.2 genes could lead to increased crop yields and potentially reduce the need for land conversion for agriculture, protecting biodiversity and natural ecosystems.

Communication benefits

the benefits of communicating this research information outweigh the risks, but only if done responsibly and transparently.

Plants

Seed stocks

The soybean (Glycine max L.) cultivars included used in this study were Young (wild type), the Cd tolerant AMT2.2 positive transgenic (T3) plants (OX), and the Cd sensitive AMT2.1/2.2 mutant plant (MU) which were generated from Young (WT).

Novel plant genotypes

To construct the CaMV35S-drived GmAMT2.2, the full-length CDS of GmAMT2.2 was cloned into the pTF101-eGFP vector, and the resultant construct was introduced into Agrobacterium tumefaciens EHA101. To produce the GmAMT2.1 and GmAMT2.2 loss-of-function mutants, the gene-editing tool CRISPR-Cas9 was used to knockout GmAMT2.1 and GmAMT2.2. CRISPR/Cas9-mediated gene editing was performed as previously reported32. Briefly, the two sgRNAs targeting the first exon of GmAMT2.1 and GmAMT2.2 were designed on CRISPR-P server (http://crispr.hzau.edu.cn/CRISPR/)33. The sgRNAs was sub-cloned into pGES201 plasmid, and the resultant construct was further introduced into Agrobacterium tumefaciens EHA105. Agrobacterium-mediated transformation as previously reported32,34, and the soybean variety Young was used as the transgene receptor.

Authentication

Total RNA was extracted from soybean or Arabidopsis thaliana with a TRNzol Universal Kit (DP424, TIANGEN, Beijing, China). The cDNA was synthesized from total RNA using a PrimeScript RT Reagent Kit with gDNA Eraser (RR047A, Takara Bio, Japan) according to the manufacturer's instructions. DNA fragment amplification was performed using KOD FX neo (TOYOBO (SHANGHAI) BIOTECH CO., Shanghai, China). qRT-PCR was conducted using TB Green Premix Ex Taq II (RR820, Takara Bio, Japan) with a CFX96 Real-Time System (Bio-Rad, Hercules, CA, USA). Data were normalized to the reference genes GmActin3. All analyses were performed with three biological replicates and three technical replicates. The results were analyzed using the 2–ΔΔCt method. Student's t-test implemented in Excel software (Excel 2016) was used to evaluate the statistical significance of the data.
